# Wogonin Attenuates Atherosclerosis via KLF11‐Mediated Suppression of PPARα‐YAP1‐Driven Glycolysis and Enhancement of ABCA1/G1‐Mediated Cholesterol Efflux

**DOI:** 10.1002/advs.202500610

**Published:** 2025-05-21

**Authors:** Chuanrui Ma, Yunqing Hua, Shu Yang, Yun Zhao, Wei Zhang, Yaodong Miao, Jing Zhang, Boxuan Feng, Guobin Zheng, Lan Li, Zhihao Liu, Han Zhang, Mingjun Zhu, Xiumei Gao, Guanwei Fan

**Affiliations:** ^1^ First Teaching Hospital of Tianjin University of Traditional Chinese Medicine National Clinical Research Center for Chinese Medicine Acupuncture and Moxibustion Tianjin 300381 China; ^2^ State Key Laboratory of Component‐Based Chinese Medicine Tianjin 301617 China; ^3^ Tianjin Key Laboratory of Translational Research of TCM Prescription and Syndrome Tianjin 300381 China; ^4^ Department of Geriatrics Peking University Shenzhen Hospital, Shenzhen, China Shenzhen Guangdong 518000 China; ^5^ Second Affiliated Hospital of Tianjin University of Traditional Chinese Medicine Tianjin 300250 P. R. China; ^6^ NHC Key Laboratory of Hormones and Development Tianjin Key Laboratory of Metabolic Diseases Chu Hsien‐I Memorial Hospital & Tianjin Institute of Endocrinology Tianjin Medical University Tianjin 300134 China; ^7^ Department of Cardiovascular Diseases The First Affiliated Hospital of Henan University of Traditional Chinese Medicine Zhengzhou 450000 China; ^8^ Haihe Laboratory of Modern Chinese Medicine Tianjin 300193 China

**Keywords:** atherosclerosis, fatty acid oxidation, KLF11, metabolic reprogramming, PPARα

## Abstract

Atherosclerosis, a chronic inflammatory disorder and leading cause of cardiovascular disease, is characterized by macrophage‐derived inflammation and foam cell formation. Emerging evidence suggests that metabolic reprogramming of macrophages represents a promising therapeutic approach for atherosclerosis management. In this study, the therapeutic potential of wogonin, a bioactive flavonoid isolated from Scutellaria baicalensis, in modulating macrophage metabolism and attenuating atherogenesis is investigated. Wogonin reduces lesion size and plaque vulnerability, accompanied by a reduction in foam cell formation and inflammation. Mechanistically, wogonin reprogrammes macrophage metabolism from glycolysis to fatty acid oxidation (FAO) by activating the PPARα‐CPT1α pathway and acts as a mitochondrial protector by activating PPARα. Wogonin also promotes the KLF11 expression and KLF11 knockout exacerbated atherosclerosis and abolishes the inhibitory effect of wogonin on glycolysis and atherosclerosis. KLF11 forms a transcriptional complex with PPARα and YAP1, serving both as a brake on PPARα‐YAP1‐mediated glycolysis and a transcriptional activator of ABCA1/G1. Collectively, wogonin reprograms macrophage metabolism from glycolysis to FAO through activation of the PPARα‐KLF11‐YAP1 pathway, thereby reducing inflammation and foam cell formation, ultimately attenuating atherogenesis.

## Graphical abstract

PPARα promotes KLF11 expression and recruits KLF11 and YAP1 to form a transcription complex, where KLF11 acts as a brake in PPARα‐YAP1‐mediated glycolysis. Wogonin reshapes the metabolism of macrophages from glycolysis to fatty acid oxidation via activation of the PPARα‐KLF11‐YAP1 pathway, by which wogonin reduces inflammation and foam cell formation and thereby attenuates atherogenesis.

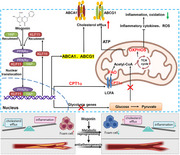

## 1 Introduction

Atherosclerosis, a chronic vascular pathology characterized by persistent inflammation and aberrant lipid accumulation, represents the principal pathological basis for major adverse cardiovascular events.^[^ ^1^ ^]^ Macrophages play a central role in all stages of atherosclerosis.^[^ ^2^ ^]^ Macrophage‐derived foam cells, a major component of atherosclerotic lesions, can result in vascular inflammation, which in turn, provide a microenvironment where macrophages are polarized toward a proinflammatory phenotype.^[^ ^3^ ^]^ Proinflammatory M1 macrophages exhibit elevated ox‐LDL uptake coupled with impaired cholesterol efflux,^[^ ^4^ ^]^ which accelerates foam cell formation. Conversely, M2 macrophages counteract disease progression through multifaceted mechanisms, including anti‐inflammatory cytokine secretion, enhanced cholesterol efflux, and promotion of necrotic cell clearance.^[^ ^2a^ ^]^ Therefore, modulating the macrophage phenotype can be a promising antiatherogenic strategy.

Metabolic reprogramming can regulate macrophage subtype and function.^[^ ^5^ ^]^ Mitochondria govern cellular metabolism through dual fuel utilization: glycolysis‐derived pyruvate and fatty acid oxidation (FAO) products converge in the TCA (tricarboxylic acid) cycle to fuel OXPHOS (oxidative phosphorylation).^[^ ^5a^ ^]^ M1 macrophages with a high level of glycolysis are accompanied by the accumulation of citric and succinic acids that disrupt the TCA cycle,^[^ ^5^, ^6^ ^]^ which drives higher fatty acid synthesis, exacerbates proinflammatory factor production, and result in inefficient ATP (adenosine triphosphate) generation accompanied by detrimental ROS (reactive oxygen species) production.^[^ ^7^ ^]^ Additionally, aberrant accumulation of fatty acids and ox‐LDL in macrophages contributes to foam cell formation and inflammatory responses.^[^ ^8^ ^]^ M2 macrophages are prone to FAO to promote ATP, and reducing inflammation,^[^ ^5^, ^9^ ^]^ suggesting that FAO is essential for M2 polarization. Pharmacological disruption of mitochondrial OXPHOS prevents M1 to M2 repolarization,^[^ ^10^ ^]^ while glycolysis inhibition mitigates M1 polarization.^[^ ^11^ ^]^ Noticeably, atherosclerotic plaques in the carotid arteries showed a heightened glycolytic activity.^[^ ^12^ ^]^ Collectively, these findings demonstrate that metabolic reprogramming acts as a dual‐target strategy to simultaneously address lipid accumulation and inflammation in atherosclerosis. Beyond energy production, mitochondria serve as integrative hubs coordinating lipid metabolism, redox balance, and inflammatory signaling.^[^ ^13^ ^]^ ABCA1 and ABCG1‐mediated cholesterol efflux can reduce cholesterol deposition in foam cells within the plaques, which exhibits strict dependence on mitochondrial ATP production,^[^ ^14^ ^]^ as evidenced by marked efflux impairment following OXPHOS inhibition.^[^ ^15^ ^]^ Pathologically, mitochondrial dysfunction in macrophages compromises cholesterol clearance and exacerbates atherogenesis.^[^ ^16^ ^]^ Moreover, ox‐LDL compromises mitochondrial homeostasis through dual mechanisms: enriching LCFAs while suppressing FAO, thereby inducing mitochondrial damage, ROS generation, and subsequent foam cell formation.^[^ ^16^ ^]^ Therefore, targeting mitochondria to reprogram macrophage metabolism is a viable therapeutic avenue for atherosclerosis.

Wogonin is a flavonoid component extracted from *Scutellaria baicalensis* which is shows an inhibitory effect on inflammation, atherosclerosis, and hyperlipidemia.^[^ ^17^ ^]^ Noticeably, wogonin attenuates inflammation,^[^ ^18^ ^]^ enhances cholesterol efflux capacity and attenuates macrophage foam cell formation,^[^ ^19^ ^]^ and inhibits apoptosis of VSMCs (vascular smooth muscle),^[^ ^20^ ^]^ which indicates that wogonin has the potential for the prevention and treatment of atherosclerosis. Intriguingly, emerging evidence positions mitochondria as a key cellular target of wogonin.^[^ ^21^ ^]^ In addition, wogonin exerts a regulatory effect on energy metabolism by inhibiting some key glycolytic enzymes, suggesting that wogonin may reprogram the metabolism.^[^ ^22^ ^]^ These studies indicate that wogonin has a potential antiatherogenic effect through regulating metabolic reprogramming. Nevertheless, the direct antiatherogenic efficacy of wogonin and its precise molecular mechanisms remain to be definitively elucidated. Therefore, we subsequently determined whether the antiatherogenic effects of wogonin and its underlying mechanism were associated with regulating metabolic reprogramming. In this study, we found that wogonin significantly attenuated atherosclerosis by reducing foam cell formation and inflammation. Notably, wogonin orchestrated metabolic reprogramming in macrophages, shifting their metabolic preference from glycolysis to FAO, thereby fueling OXPHOS and preserving mitochondrial integrity via coordinated activation of PPARα and KLF11. Mechanistically, PPARα recruited YAP1 and KLF11 to assemble a functional transcription complex. Within this complex, KLF11 suppressed the YAP1‐mediated glycolysis and activated the PPARα‐mediated FAO. Furthermore, KLF11 transactivates ABCA1/G1 expression, thereby enhancing cholesterol efflux capacity and mitigating foam cell formation. Genetic validation studies established KLF11's pivotal role in wogonin's therapeutic effects. KLF11 knockout abolished the inhibitory effect of wogonin on glycolysis, thereby exacerbating atherosclerosis and reducing the antiatherogenic effect of wogonin. In contrast, KLF11 restoration in KLF11^−/−^ mice attenuated atherosclerosis and enhanced plaque stability via reducing glycolysis. Collectively, wogonin reshaped the metabolism of macrophages from glycolysis to FAO via activation of the PPARα‐KLF11‐YAP1 pathway, by which wogonin reduced inflammation and foam cell formation and thereby attenuated atherogenesis. These discoveries position KLF11 as a central metabolic regulator in atherogenesis while establishing wogonin as a promising metabolic modulator for cardiovascular therapeutics.

## 2 Results

### 2.1 Wogonin Attenuated Atherosclerosis and Enhanced Plaque Stability

**Figure 1:**
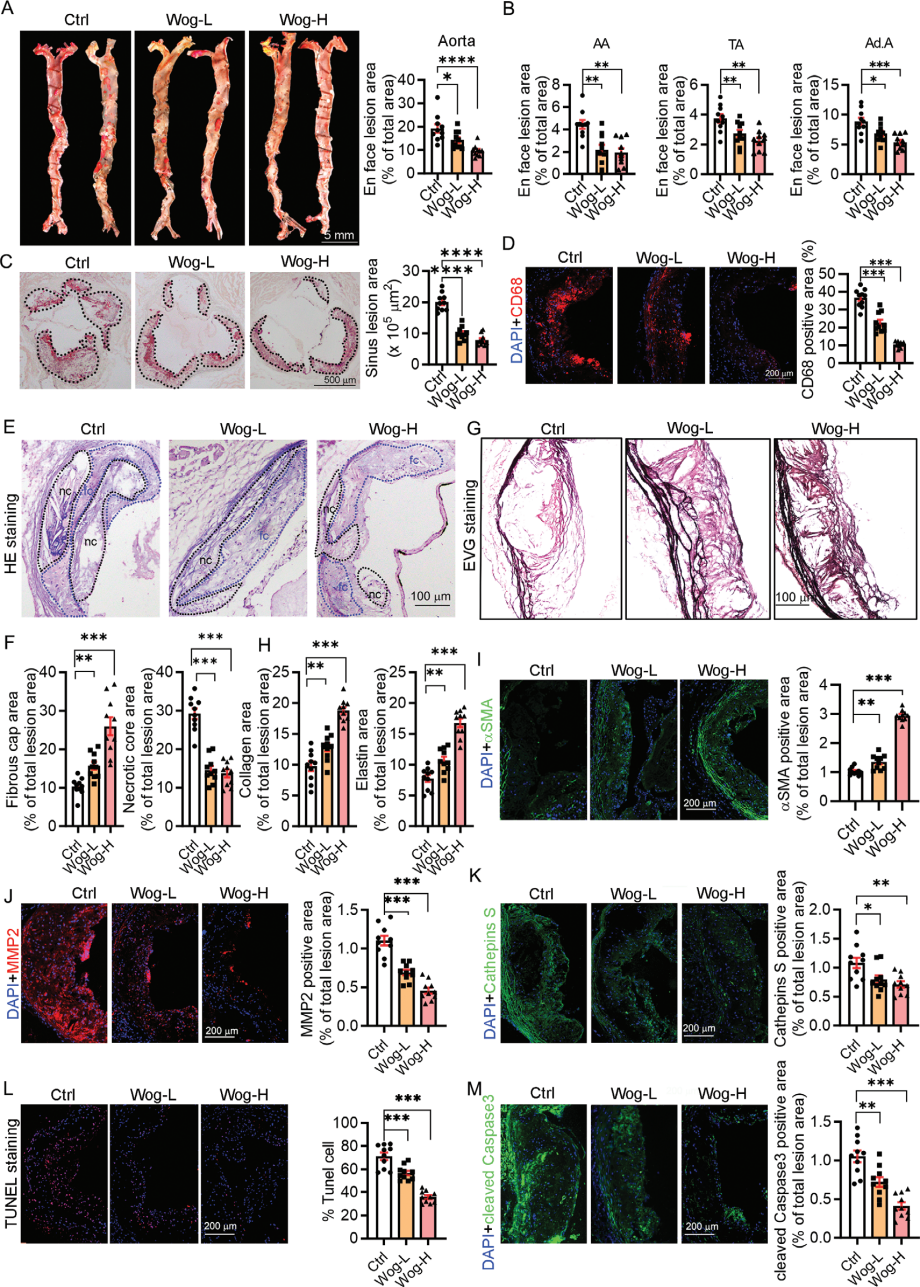
Wogonin attenuated atherosclerosis and reduced plaque vulnerability. A,B) The aortas were isolated for the determination of lesions in en face aortas by Oil Red O staining and were quantified by a computer‐assisted image analysis protocol. Lesion areas in whole aorta, AA, TA, and Ad.A was expressed as a ratio of lesion area to total aorta area. AA: aortic arch; TA: thoracic aorta; Ad.A: abdominal aorta; *n* = 10. Scale bar: 5 mm. C) Representative photomicrographs and quantification of plaque in aortic root sections stained with Oil Red O solution. The aortic lesion area was expressed in µm^2^, *n* = 10. Scale bar: 500 µm. D) Immunostaining for CD68 and quantification was expressed as the ratio of positive areas to plaque area, *n* = 10. Scale bar: 200 µm. E,F) Hematoxylin and eosin staining was followed by quantitative analysis of the necrotic core area and fibrous cap area in the aortic root. nc: necrotic cores marked by a black dotted line; fc: fibrous cap marked by a blue dotted line, *n* = 10. Scale bar: 100 µm. G,H) Collagen (red) and elastin (black) were quantitatively analyzed following EVG staining, *n* = 10. Scale bar: 100 µm. Immunostaining for α‐SMA I), MMP‐2 J), and cathepin S K) and quantification was expressed as the ratio of positive areas to plaque area, *n* = 10. Scale bar: 200 mm. L) TUNEL staining for cellular apoptosis in the plaque and quantification of positive areas, *n* = 10. Scale bar: 200 µm. M) Detection of intraplaque cleaved‐caspase3 levels by immunofluorescence staining, *n* = 10. Scale bar: 200 µm. Data are presented as mean ± SEM. *p*‐values are shown in the figure A, B, C, D, F, H, and I–M) by One‐way ANOVA with Tukey's multiple comparisons test. *p < 0.05, **p < 0.01, ***p < 0.001, and ****p < 0.0001, significantly different from the Ctrl. Ctrl: control; Wog‐L: wogonin in low dose; Wog‐H: wogonin in high dose.

An initial assessment confirmed that administration of wogonin had no significant effect on blood pressure, heart rate, peripheral blood cell counts, or food intake in mice at baseline (Figure S1 and Table S2, Supporting Information). After treatment with high‐fat diet (HFD) containing wogonin for 16 weeks, the atherosclerotic lesion size in the aorta and aorta root from LDLR^−/−^ mice was assessed by Oil red O staining. Wogonin significantly reduced the lesion area in both the en face aorta and aorta root (**Figure** **1**A–C). Notably, high doses of wogonin reduced body weight and liver‐to‐body ratio in LDLR^−/−^ mice, concurrent with ameliorated fatty liver as well as reduced hepatocyte ballooning and lipid droplet deposition, lowered serum aspartate aminotransferase (AST) levels (Figure S2, Supporting Information), and improved lipid profiles (Figure S3, Supporting Information). In addition, the foam cells, the hallmark of atherosclerosis, within the atherosclerotic plaque were markedly reduced by wogonin, which was indicated by immunostaining with marker CD68 (Figure 1D). These data suggest that wogonin can attenuate atherogenesis and improve lipid metabolism. To further determine whether wogonin can reduce plaque vulnerability, we assessed the composition of plaque by different types of staining. Intriguingly, HE staining showed that wogonin reduced the necrotic core area and increased the fibrous cap in the plaque (Figure 1E,F). Furthermore, wogonin significantly increased collagen and elastin content within plaques (Figure 1G,H) and elevated VSMCs density while suppressing VSMC apoptosis (Figure 1I; and Figure S4A, Supporting Information). MMPs are a family of zinc‐dependent proteolytic enzymes that degrade various components of the extracellular matrix, mediate tissue remodeling, and facilitate extracellular matrix breakdown during atherogenesis.^[^ ^23^ ^]^ Cathepsins are specifically localized within lysosomes and endosomes, where they function to degrade unnecessary intracellular proteins.^[^ ^24^ ^]^ Under the pathological conditions of atherosclerosis, infiltrating inflammatory macrophages release cathepsins into plaques, leading to degradation of the extracellular matrix and subsequent promotion of plaque rupture.^[^ ^25^ ^]^ Wogonin reduced the levels of MMP‐2/9 (Figure 1J; and Figure S4B, Supporting Information) and cathepsins S/K in plaques (Figure 1K; and Figure S4C, Supporting Information), which may contribute to the restoration of collagen fibers and enhanced plaque stability. Apoptosis analysis further revealed that wogonin significantly reduced apoptotic cells within the plaques (Figure 1L), which was accompanied by reduced cleaved‐caspase‐3 expression levels (Figure 1M). Altogether, these findings suggested that wogonin attenuated atherosclerosis and increased plaque stability.

### 2.2 Wogonin Exerted the Antiatherogenic Effect Partially via Reducing Inflammation In Vivo and In Vitro

**Figure 2:**
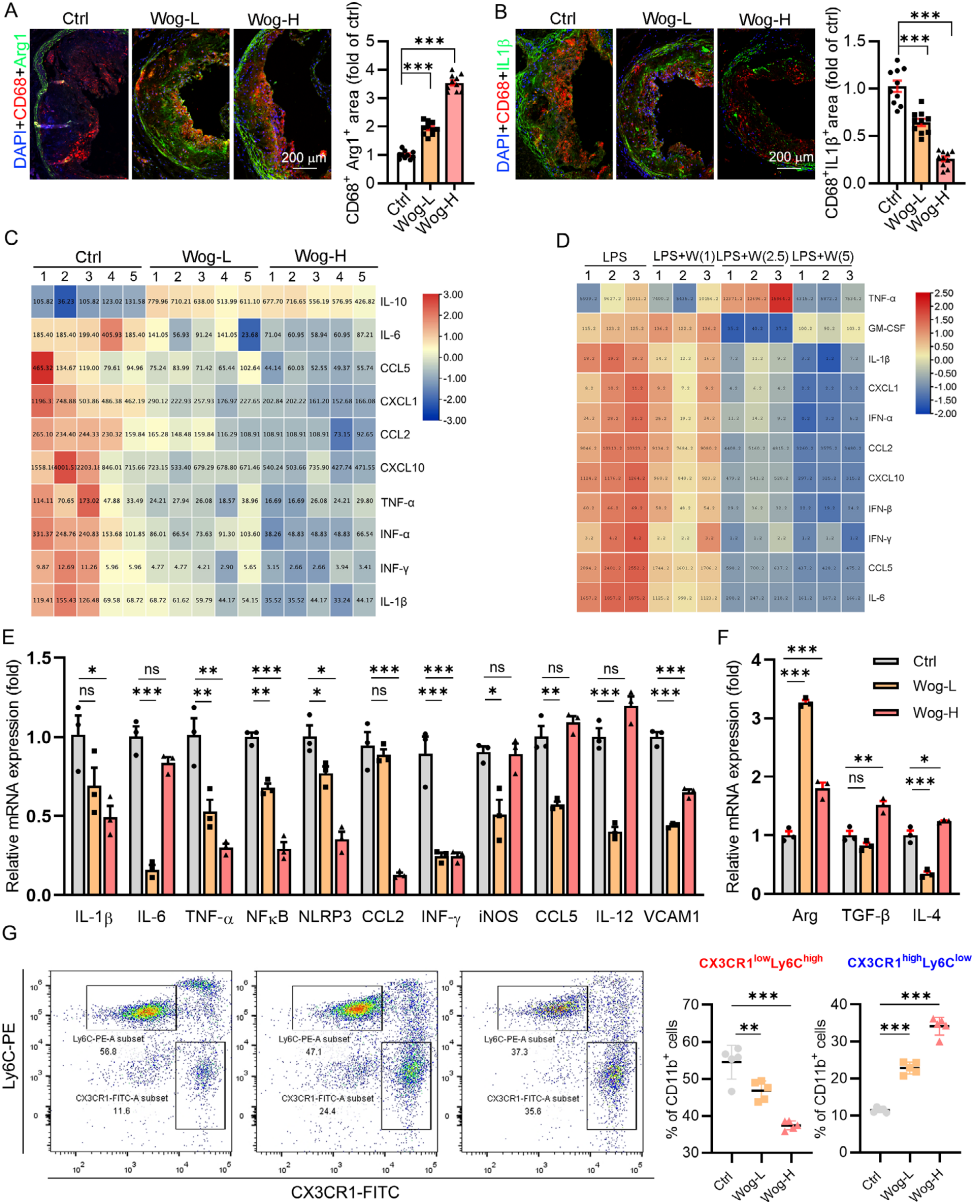
Wogonin reduced inflammation in vitro and in vivo. A,B) Aortic root cross sections were performed with coimmunofluorescent staining with anti‐CD68 and Arg1 or IL1β antibodies with quantitative analysis of CD68^+^Arg^+^ or CD68^+^IL1β^+^ area, *n* = 10, Scale bar: 200 µm. C,D) Inflammatory cytokines in the serum and culture medium were detected by the LEGENDplexTM Mouse Anti‐Virus Response Panel, *n* = 5 in vivo, and *n* = 3 in vitro. E,F) The expression of proinflammatory and anti‐inflammatory cytokine mRNA in macrophages was determined by q‐RT‐PCR, *n* = 3. G) Two subpopulations of the monocytes that were characterized as Ly6C^high^CX3CR1^low^ and Ly6C^low^CX3CR1^high^ were detected by flow cytometry, *n* = 5. *p*‐values are shown in the figure A,B,E,F) by One‐way ANOVA with Dunnett's multiple comparisons or One‐way ANOVA with Tukey's multiple comparisons test G). Data are presented as mean ± SEM. *p < 0.05, **p < 0.01, and ***p < 0.001, significantly different as indicated; ns: not significantly different.

Inflammation contributes to atherogenesis. In this study, the expression of anti‐inflammatory cytokine Arg1 was elevated while the expression of proinflammatory cytokine IL1β was decreased in the plaque macrophages after wogonin treatment (**Figure** **2**A,B). LDL functions as a relevant autoantigen inducing autoimmune response in the progression of atherosclerosis.^[^ ^26^ ^]^ Within the plaque, interactions between antigen‐presenting cells, such as macrophages and T cells further exacerbate proinflammatory cytokine secretion.^[^ ^27^ ^]^ Consequently, reducing T cell infiltration represents a strategic approach to mitigate chronic inflammatory diseases.^[^ ^28^ ^]^ Our findings demonstrated wogonin decreased T cell accumulation in plaques (Figure S4D, Supporting Information). Accordingly, wogonin markedly reduced the inflammatory response in vivo, evidenced by the lower level of inflammatory cytokines in serum and in the peritoneal macrophage from wogonin‐treated LDLR^−/−^ mice compared to the control group (Figure 2C,E,F). In vitro, the level of inflammatory cytokines in the medium of RAW264.7 cells treated with wogonin in the presence of LPS was decreased, while the anti‐inflammatory cytokines were increased (Figure 2D). The circulating Ly6C^high^ proinflammatory monocyte subpopulation is the predominant subpopulation that gives rise to plaque macrophages during atherogenesis.^[^ ^29^ ^]^ We then analyzed the monocyte subtypes in the blood of HFD‐fed LDLR^−/−^ mice. Two subpopulations of the monocytes were characterized as Ly6C^high^CX3CR1^low^ and Ly6C^low^CX3CR1^high^. We found that the number of proinflammatory Ly6C^high^CX3CR1^low^ monocytes was lower in the blood of wogonin‐treated mice as compared with that of the control mice (Figure 2G). In contrast, the proportion of anti‐inflammatory Ly6C^low^CX3CR1^high^ monocytes in the blood of wogonin‐treated mice was higher than those in control mice (Figure 2G). These data indicated that wogonin promoted monocytes/macrophages toward an anti‐inflammatory phenotype. Taken together, wogonin significantly reduced the inflammation in vivo and in vitro, partially by which wogonin attenuated atherogenesis.

### 2.3 Wogonin Inhibited Foam Cell Formation by Enhancing Cholesterol Efflux In Vitro and In Vivo

**Figure 3:**
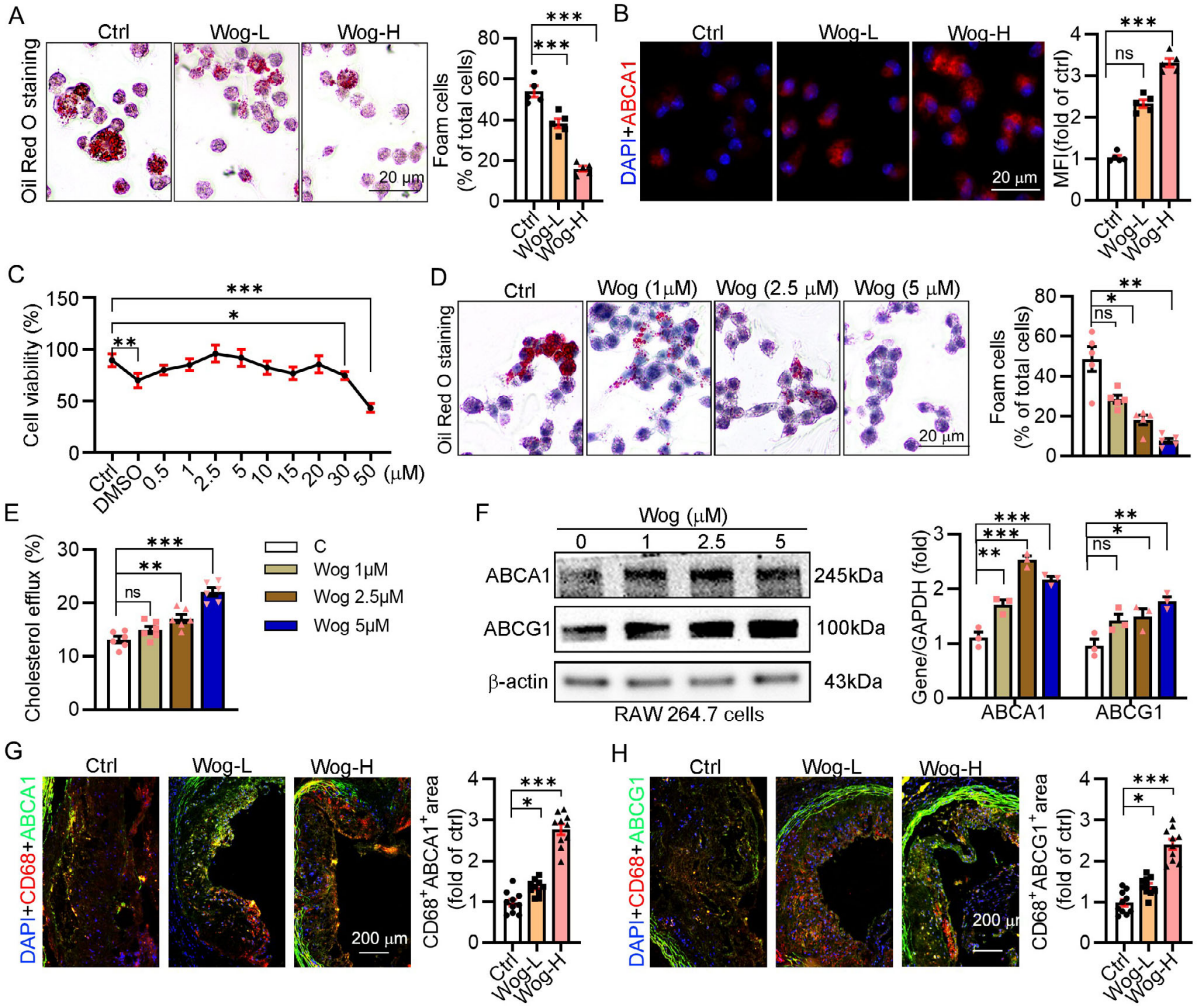
Wogonin inhibited lipid accumulation in macrophages by promoting cholesterol efflux. A) Oil red O staining on peritoneal macrophages from wogonin‐treated mice to assess the formation of foam cells, *n* = 5. Scale bar: 20 µm. B) Detection of the ABCA1 expression in peritoneal macrophages by immunofluorescence staining, *n* = 5. Scale bar: 20 µm. C) The MTT assay kit was used to test the effect of wogonin on cell viability at different concentrations, *n* = 6. D) RAW264.7 cells were incubated with ox‐LDL to induce the formation of foam cells in the presence of wogonin and subsequently stained with Oil red O, *n* = 5. Scale bar: 20 µm. E) A cholesterol efflux assay was conducted after treatment with wogonin, *n* = 6. F) Detection of the ABCA1 and ABCG1 expression in RAW264.7 cells by Western Blot after treatment with wogonin, *n* = 3. G,H) Coimmunofluorescent staining with anti‐CD68 and Arg1 or IL1β antibodies with quantitative analysis of CD68^+^Arg^+^ or CD68^+^IL1β^+^ area, *n* = 10. Scale bar: 200 µm. Data are presented as mean ± SEM, *p*‐values are shown in the figure A–H) by One‐way ANOVA with Tukey's multiple comparisons test. *p < 0.05, **p < 0.01, and ***p < 0.001, significantly different as indicated.

Macrophage‐derived foam cell in atherosclerotic lesions plays a vital role in the progress of atherosclerosis. To further determine the inhibitory effect of wogonin on foam cell formation, we detected lipid deposition in the macrophages. As expected, wogonin markedly reduced the lipid accumulation in the macrophages in vivo (**Figure** **3**A). The cellular cholesterol content was determined by the level of cholesterol efflux and uptake, which was controlled by the transporters ABCA1 and ABCG1, as well as the scavenger receptors.^[^ ^30^ ^]^ Wogonin increased the expression of ABCA1 in the peritoneal macrophages from the LDLR^−/−^ mice (Figure 3B). Moreover, we evaluated the toxicity of wogonin on cells before the in vitro experiment. Wogonin did not affect the cell viability of RAW264.7 cells before the working concentration reached 30 µm (Figure 3C). In addition, wogonin markedly reduced the lipid accumulation in ox‐LDL‐treated RAW264.7 cells, indicating that wogonin inhibited foam cell formation in vitro (Figure 3D). In line with this inhibitory effect on the foam cells, the level of cholesterol efflux as well as the expression of ABCA1 and ABCG1 was increased after wogonin treatment (Figure 3E,F), whereas the uptake of ox‐LDL and the expression of scavenger receptors that orchestrating ox‐LDL uptake was not affected by wogonin treatment (Figure S5, Supporting Information). Furthermore, we detected the expression of ABCA1 and ABCG1 in the plaque macrophages. Correspondingly, wogonin increased the expression of ABCA1 and ABCG1 in the plaque macrophages (Figure 3G,H). Taken together, wogonin inhibited foam cell formation by enhancing the ABCA1‐ and ABCG1‐mediated cholesterol efflux.

### 2.4 Wogonin Upregulated CPT1α Expression via Binding and Stimulating PPARα Nuclear Translocation

**Figure 4:**
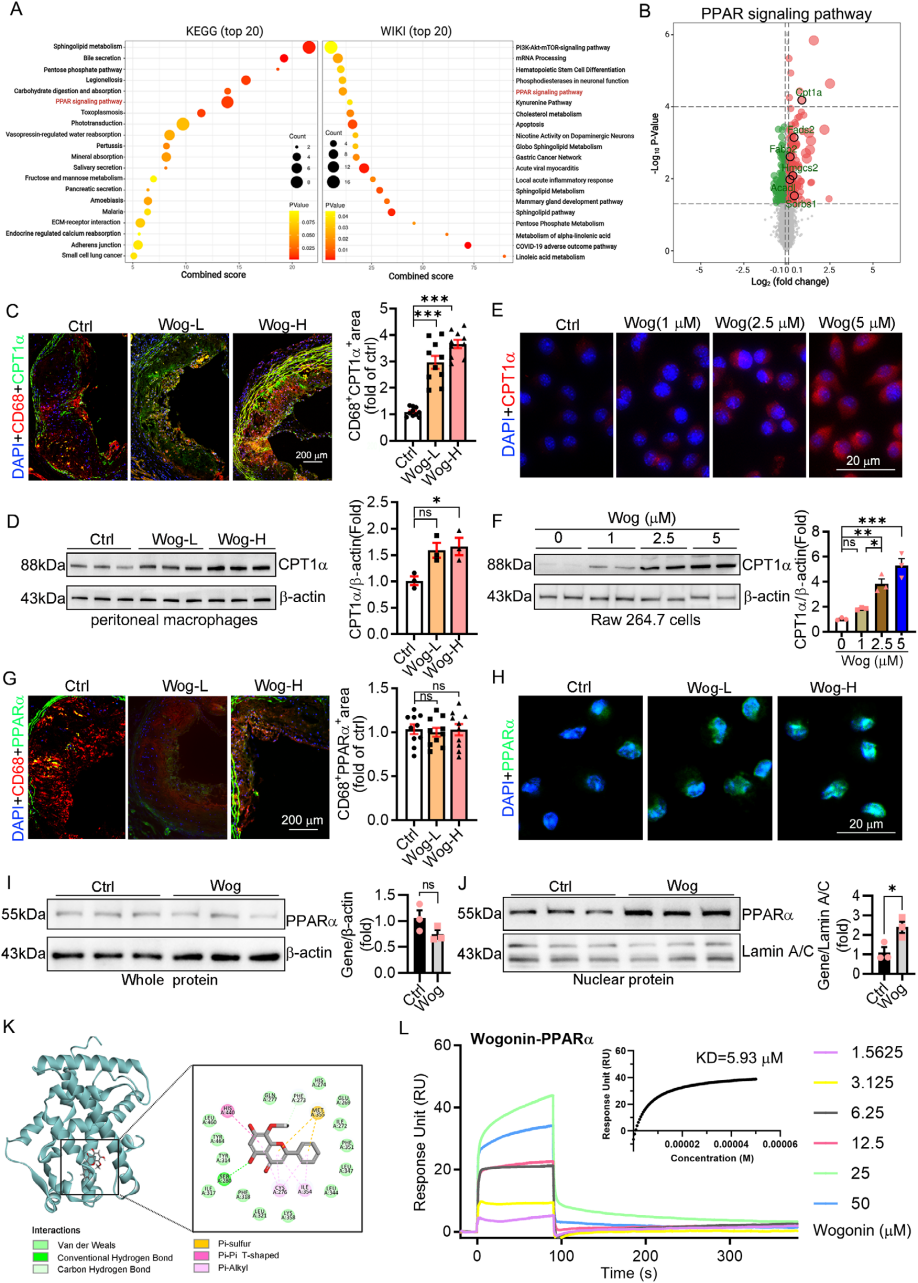
Wogonin activated CPT1α expression by binding and promoting PPARα nuclear translocation. A) Gene ontology (GO) terms and Kyoto Encyclopedia of Genes and Genomes (KEGG) pathways enriched in differentially expressed genes in macrophages after wogonin treatment. B) Transcriptome analysis of PPAR signaling in wogonin‐treated peritoneal macrophages. log_2_‐based transformation on ratio fold change values was used, where red shading indicates upregulation and green indicates down‐regulation. For each group, *n* = 3. C) The protein level of CPT1α in the plaque macrophages from mice treated with wogonin was determined by coimmunostaining with CD68 and CPT1α, *n* = 10. Scale bar: 200 µm. D) The protein level of CPT1α in peritoneal macrophages from mice treated with wogonin was determined by western blotting, *n* = 3. E,F) The levels of CPT1α in RAW264.7 cells after wogonin treatment were detected by immunofluorescence staining (*n* = 10, Scale bar: 20 µm) and western blotting (*n* = 3). G,H) Nuclear displacement of PPARα was detected by immunofluorescence staining in plaque macrophages (*n* = 10, Scale bar: 200 µm) and peritoneal macrophages (*n* = 3, Scale bar: 20 µm). I,J) The expression of PPARα in whole and nuclear proteins of RAW264.7 cells was detected by western blotting after treatment with wogonin, *n* = 3. K) Molecular docking predicts the presence of binding targets for wogonin and PPARα to form stable compounds. L) Surface plasmon resonance assay for interaction of wogonin was passed over the Biacore chip surfaces immobilized with recombinant PPARα protein: Biacore diagram and estimated dissociation constant value (KD) for wogonin binding PPARα. Data are presented as mean ± SEM. *p*‐values are shown in the figure C,D,F,G,I,J) by One‐way ANOVA with Tukey's multiple comparisons test. *p < 0.05, **p < 0.01, and ***p < 0.001, significantly different as indicated.

To systemically explore the mechanism by which wogonin protected against atherosclerosis, we performed RNA‐sequencing (RNA‐seq) to investigate the transcriptional profile of associated genes with wogonin treatment. RNA‐seq data showed that wogonin markedly activated the PPAR signaling pathway that plays a critical role in regulating cellular metabolism (**Figure** **4**A). The downstream genes of PPARα were upregulated in mRNA levels after wogonin treatment, with the most significant increase in CPT1α (Figure 4B). CPT1α is a key gene in fatty acid metabolism and is localized to the outer mitochondrial membrane.^[^ ^31^ ^]^ Fatty acids are transported into the mitochondrion via CPT1α to participate in β‐oxidation in macrophages, which can inhibit atherosclerosis progression.^[^ ^32^ ^]^ Next, we investigated whether wogonin affects the activity or expression of CPT1α in the plaque and in vitro. We detected the protein level of CPT1α in the plaque macrophages by coimmunostaining and found that the expression of CPT1α in plaque macrophages and peritoneal macrophages was markedly increased (Figure 4C,D). Moreover, in vitro, wogonin markedly increased the expression of CPT1α in RAW264.7 cells (Figure 4E,F). PPARα is the upstream regulator of CPT1α.^[^ ^33^ ^]^ However, we found that wogonin did not affect the expression of PPARα (Figure 4G), but stimulated its nuclear translocation (Figure 4H), which was supported by western blot in wogonin‐treated Raw264.7 cells (Figure 4I,J). Noticeably, wogonin did not affect the expression and nuclear translocation of PPARβ and PPARγ (Figure S6A,B, Supporting Information). Moreover, the docking assay to predict the binding between wogonin and PPARα showed that wogonin also has the potential for binding with PPARα (Figure 4K). We further used surface plasmon resonance measurements to confirm the direct binding of wogonin and PPARα, and the estimated dissociation constant value (KD) was 5.93 µm (Figure 4L). Taken together, wogonin stimulates the PPARα signaling pathway and upregulates the level of CPT1α by binding and promoting its nuclear translocation.

### 2.5 Inhibition of CPT1α or PPARα Exacerbated Atherogenesis and Abolished the Antiatherogenic Effect of Wogonin

**Figure 5:**
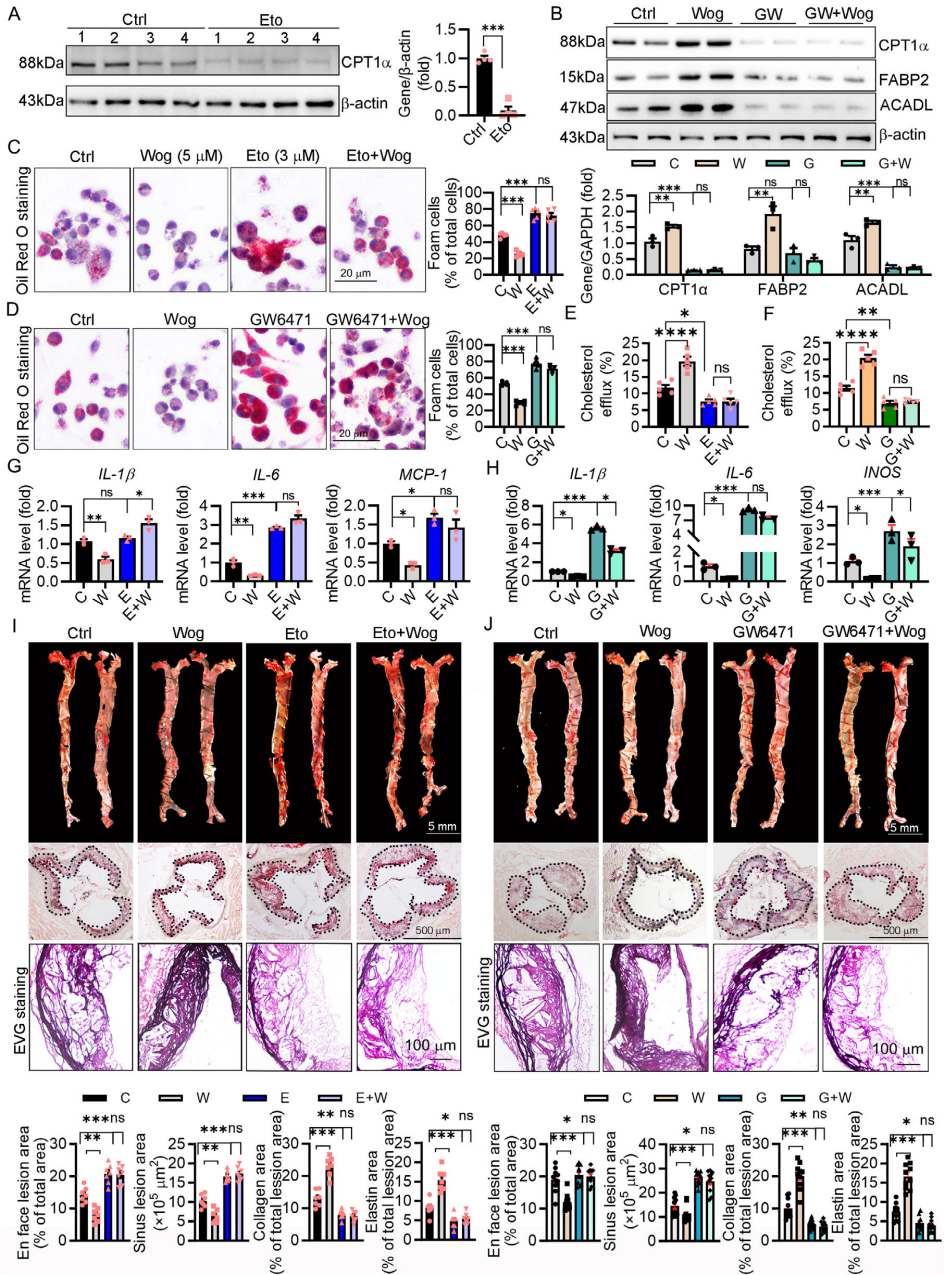
Antiatherogenic effects of wogonin was reliant on activating PPARα‐CPT1α‐mediated inhibition of foam cell formation and inflammation. A) The expression of CPT1α in the protein of etomoxir‐treated RAW264.7 cells was determined by western blotting, followed by quantification, *n* = 4, unpaired Student's *t*‐test. B) CPT1α, FABP2, and ACADL in RAW264.7 cells were evaluated by western blotting after indicated treatment, *n* = 3. C,D) In the presence of etomoxir or GW6471 and/or wogonin, RAW264.7 cells were incubated with ox‐LDL for 24 h to induce foam cell formation, subsequently stained with Oil Red O, *n* = 5. Scale bar: 20 µm. E,F) Cholesterol efflux levels were assessed by measuring the fluorescence intensity of cell supernatants and lysates after incubation with 3‐dodecanoyl‐NBD cholesterol, *n* = 5. G,H) RAW264.7 cells were pretreated with etomoxir or GW6471 for 48 h and then treated with or without wogonin for 16 h, after which inflammatory factors were measured by qRT‐PCR, *n* = 3. I,J) HFD‐fed LDLR^−/−^ mice were treated with etomoxir (*n* = 7) or GW6471 (*n* = 10) in the presence or absence of wogonin for 16 weeks. Determination of lesions in en face aortas (Scale bar: 5 mm) and aortic root was by Oil Red O staining (Scale bar: 500 µm) and quantified by a computer‐assisted image analysis protocol. Lesion areas were expressed as the ratio of lesion area to aorta area or µm^2^. Collagen (red) and elastin (black) were quantitatively analyzed following EVG staining, Scale bar: 100 µm. Data are presented as mean ± SEM. *p*‐values are shown in the figure B–J) by One‐way ANOVA with Tukey's multiple comparisons test. *p < 0.05, **p < 0.01, and ***p < 0.001, significantly different as indicated; ns: not significantly different.

To further explore the molecular mechanism of wogonin, we used CPT1α inhibitor etomoxir or PPARα antagonists GW6471 to treat macrophages. Coherently with previous studies,^[^ ^34^ ^]^ significant reduction in CPT1α protein was achieved after etomoxir treatment (**Figure** **5**A). Likewise, the PPARα antagonist significantly reduced the expression of PPARα and its downstream genes and reversed the wogonin‐induced upregulation of downstream genes of PPARα (Figure 5B). Of interest, the abrogation of CPT1α or PPARα abolished the inhibitory effect of wogonin on foam cell formation, inflammation, and the increased cholesterol efflux in vitro (Figure 5C–H). These data suggested that PPARα‐CPT1α signaling plays an important role in the inhibitory effect of wogonin on foam cell formation and inflammation. To further validate the key role of PPARα‐CPT1α in mediating the wogonin‐induced antiatherogenic effects, we treated LDLR^−/−^ mice with the etomoxir or GW6471 in the presence or absence of wogonin. Pharmacological inhibition of CPT1α or PPARα exacerbated atherogenesis by increasing plaque area and reducing collagen/elastin content (indicative of plaque destabilization), while concurrently abolishing the antiatherogenic effects of wogonin (Figure 5I,J). Moreover, inhibition of PPARα elicited M1 subtypes and abolished the effect of wogonin inducing macrophage polarization toward M2 type in vitro (Figure S7, Supporting Information). Furthermore, inhibition of PPARα promoted macrophage‐derived foam cell formation and led the macrophages and monocytes toward inflammatory status in GW6471‐treated mice (Figure S8, Supporting Information). Taken together, these findings demonstrate that wogonin exerted its antiatherogenic effects, including suppression of inflammatory responses and foam cell formation, through activation of the PPARα‐CPT1α signaling axis.

### 2.6 Wogonin Triggered Metabolic Reprogramming and Acted as a Mitochondrial Protector by Activating PPARα

**Figure 6:**
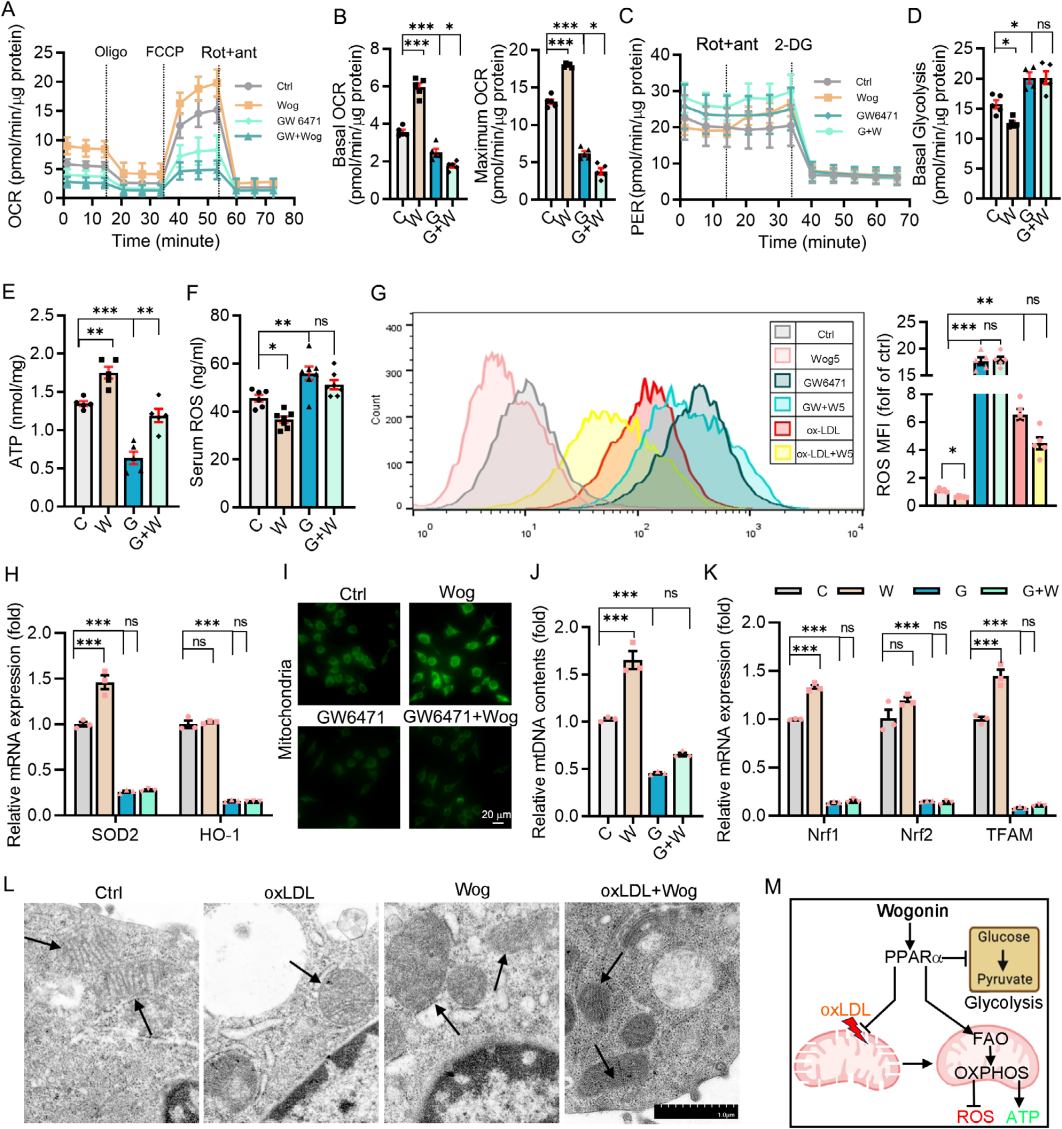
Wogonin reprogrammed the metabolism of macrophages and protected mitochondria. A) OCR curves of peritoneal macrophages from various groups of mice, *n* = 5. B) Basal and maximum oxygen consumption were normalized by protein content in each 96‐well sample, *n* = 5, One‐way ANOVA with Tukey's multiple comparisons. C,D) Tracking and quantifying the proton efflux rate and basal glycolytic capacity of peritoneal macrophages, *n* = 5, One‐way ANOVA with Tukey's multiple comparisons. E) The level of ATP in the peritoneal macrophages of each group of mice, normalized by protein, n = 5, One‐way ANOVA with Tukey's multiple comparisons. F) Serum ROS levels in mice fed wogonin and/or GW6471 were detected by the kit, *n* = 6. G) Intracellular ROS levels were detected by flow cytometry after treatment with wogonin, ox‐LDL, or GW6471, *n* = 5, Welch ANOVA with Dunnett T3's multiple comparisons. H) RAW264.7 cells were starved for 16 h and then treated with wogonin and/or GW6471 for 24 h. SOD2 and OH‐1 levels were detected by qRT‐PCR, *n* = 3, One‐way ANOVA with Tukey's multiple comparisons. I) Mitochondrial tracing by MitoTracker Green FM dye, *n* = 5. (Scale bar: 20 µm) J) mtDNA levels in treated RAW264.7 cells, *n* = 3. K) mRNA levels of mitochondrial biogenesis‐related genes Nrf1, Nrf2, and TFAM in RAW264.7 cells, *n* = 3, One‐way ANOVA with Tukey's multiple comparisons. L) Examples of electron microscopy images of peritoneal macrophages treated with wogonin or 100 µg mL^−1^ ox‐LDL for 24 h. Mitochondria with abnormal cristae structures were highlighted by black arrows, Scale bar = 1 µm. M) Schematic of wogonin biasing the metabolic mode of macrophage from glycolysis toward FAO‐OXPHOS. Data are presented as mean ± SEM. *p*‐values are shown in the figure A–J) by One‐way ANOVA with Tukey's multiple comparisons test. *p < 0.05, **p < 0.01, and ***p < 0.001, significantly different as indicated; ns: not significantly different.

Macrophage subtype and function can be profoundly altered by the switch of cellular metabolism. PPARα, as an upstream regulator of CPT1α, not only promotes FAO but also acts as a transcription factor that broadly influences multiple aspects of glucose and lipid metabolism.^[^ ^35^ ^]^ In the seahorse assay, measurements for oxygen consumption rates showed that wogonin increased the basal and maximal respiration in peritoneal macrophages with improved cellular spare respiratory capacity, and enhanced the ability of mitochondria to generate ATP to meet cellular energy requirements (**Figure** **6**A,B,E; and Figure S9, Supporting Information), indicating that wogonin promoted OXPHOS. Glycolytic rates tests were performed on peritoneal macrophages in the presence of OXPHOS inhibition by Rot/AA. Notably, peritoneal macrophages from wogonin group mice showed an increased proton efflux rate and reduced basal glycolysis levels (Figure 6C,D). These data corroborate that wogonin biased the metabolic mode of macrophage from glycolysis toward FAO‐OXPHOS in vivo and in vitro. Functionally, the reprogramming of cellular metabolism restructured the ratio between M1 and M2‐type macrophages to support an anti‐inflammatory phenotype (Figure S8C, Supporting Information). In the atherosclerotic pathological environment, accumulated ox‐LDL reprogrammed the metabolism from OXPHOS to glycolysis, promoting inflammation and oxidative stress.^[^ ^16^ ^]^ Disruption of OXPHOS leads to a massive buildup of succinate that can lead to the production of mitochondrial ROS and stabilizes HIF‐1α which continues to drive inflammation and glycolysis.^[^ ^36^ ^]^ The results suggest that wogonin reduced ROS levels in vivo and in vitro, which was abrogated by GW6471 (Figure 6F,G). Disproportionation of ROS via SOD2 in the mitochondrial matrix eliminates ROS.^[^ ^37^ ^]^ Our results indicated that wogonin promoted the transcription of redox system genes, including SOD2 and HO‐1 (Figure 6H). The occurrence of OXPHOS is dependent on the structural and functional integrity of mitochondria, and mitochondrial biogenesis is a necessary part of mitochondrial repair.^[^ ^38^ ^]^ Mitochondrial tracer tests showed that wogonin increased the number of mitochondria (Figure 6I,J) and promoted the transcription of the genes supporting mitochondrial biogenesis, including Nrf1, Nrf2, and TFAM (Figure 6K). In addition, transmission electron microscopy indicated that wogonin protected against ox‐LDL‐induced damage in cristae structures (Figure 6L). Taken together, wogonin lead metabolic switch from glycolysis to mitochondrial OXPHOS and improves mitochondrial structure in a PPARα‐dependent manner (Figure 6M).

### 2.7 KLF11 Acted as a Suppressor in Atherogenesis by Antagonizing YAP1‐Mediated Glycolysis and Wogonin Promoted the Interaction Between KLF11‐YAP1‐PPARα

**Figure 7:**
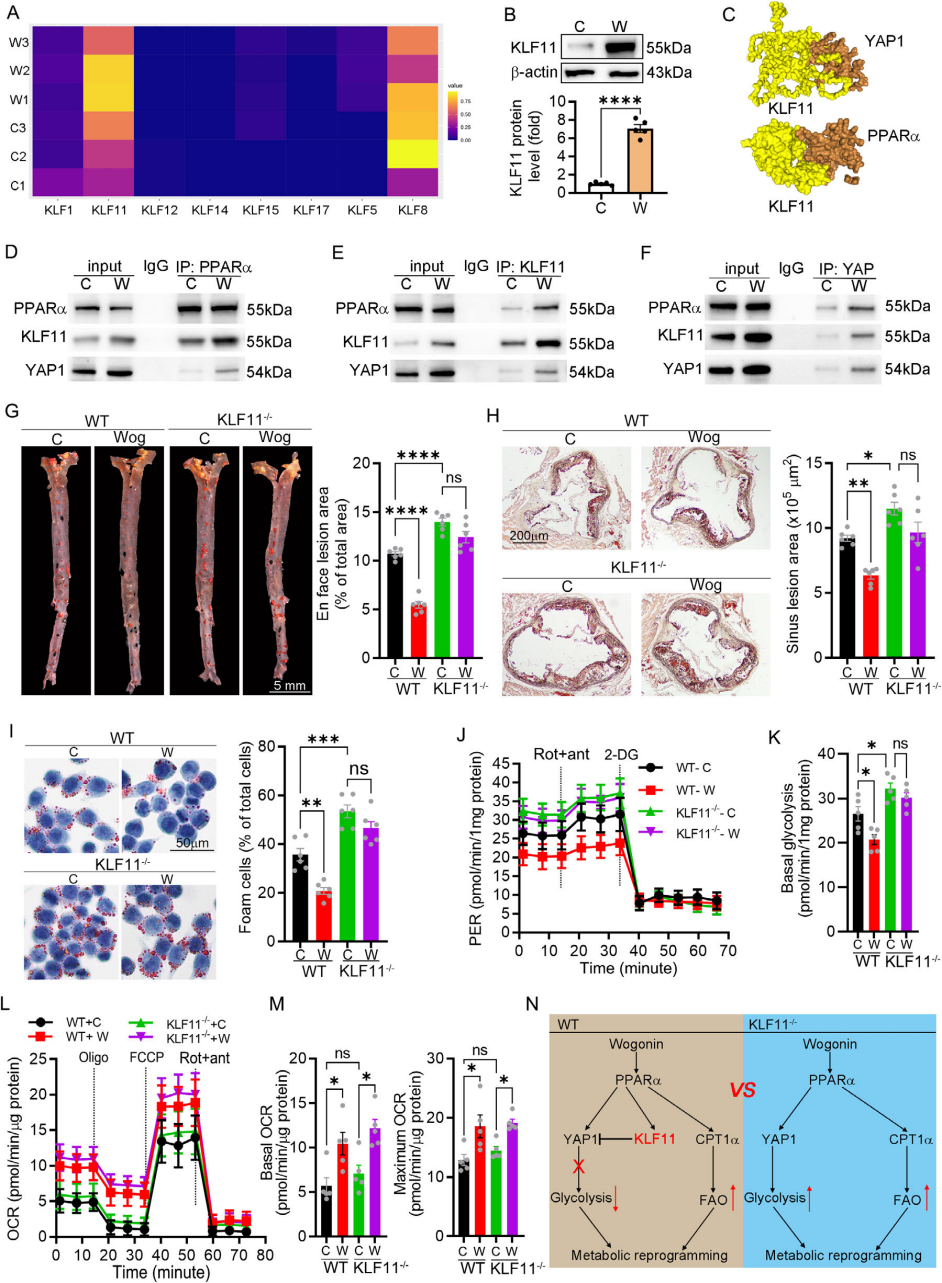
Interaction between KLF11‐YAP1‐PPARα regulated the metabolic reprogramming and the antiatherogenic effect of wogonin. A) Wogonin promoted the expression of KLF11, *n* = 5. B) Increased expression of KLF11 by wogonin was determined by western blots, *n* = 5. C) Molecular docking with the HDOCK server predicts the binding potential between KLF11 and PPARα or YAP1. (http://hdock.phys.hust.edu.cn). D–F) The interaction between KLF11, YAP1, and PPARα was determined by immunoprecipitation, *n* = 5. G,H) The antiatherogenic effect of wogonin was determined in KLF11‐deficient mice, *n* = 6. (G, Scale bar: 5 mm. H, Scale bar: 200 µm). I) Determination of foam cells by Oil red O staining in KLF11 knockout macrophages, *n* = 6. Scale bar: 50 µm. J) PER curves of peritoneal macrophages from WT and KLF11^−/−^ mice in the presence or absence of wogonin, *n* = 6. K) Basal and maximum oxygen consumption were normalized by protein content in each 96‐well sample, *n* = 5. L,M) Tracking and quantifying the proton efflux rate and basal glycolytic capacity of peritoneal macrophages from WT and KLF11^−/−^ mice in the presence or absence of wogonin, *n* = 5. N) Schematic of the role of KLF11 as a negative feedback regulator of YAP1 signaling after PPARα activation. Data are presented as mean ± SEM. *p*‐values are shown in the figure B) by two‐tailed *t*‐test or One‐way ANOVA with Tukey's multiple comparisons test G–M). *p < 0.05, **p < 0.01, ***p < 0.001, and ****p < 0.0001, significantly different as indicated; ns: not significantly different.

KLF11 were reported as putative PPARα targets.^[^ ^39^ ^]^ In this study, RNA‐seq suggested that wogonin promoted the expression of KLF11 in the transcript, which was further validated in translation by western blot (**Figure** **7**A,B). Previous studies showed that YAP1 can bind with the PPARα.^[48]^ Molecular docking analyses using the HDOCK server predicted the binding affinities between KLF11 and both PPARα and YAP1(Figure 7C), suggesting the potential formation of a PPARα‐YAP1‐KLF11 transcriptional regulatory complex. Furthermore, the immunoprecipitation assay indicated that KLF11 bound to YAP1 and PPARα (Figure 7D–F), suggesting that PPARα, YAP1, and KLF11 can form a transcription complex. The binding of YAP1 with the PPARα can induce nuclear translocation of YAP1, and YAP1 can promote the glycolysis.^[^ ^40^ ^]^ However, in this study, wogonin markedly activated PPARα, but decreased the glycolysis, indicating other regulators can suppress YAP1‐mediated glycolysis. The previous study reported that KLF11 can suppress the YAP1 signaling pathway by recruiting YAP1.^[^ ^41^ ^]^ These studies indicated that KLF11 may act as a brake in the PPARα‐YAP1 signaling pathway, which plays a critical role in the antiatherogenic effect of wogonin via regulating metabolic switch. To further validate the hypothesis, we evaluated the antiatherogenic effect of wogonin in KLF11‐deficient mice (Figure S10, Supporting Information) and observed that KLF11 knockout not only exacerbated the atherogenesis but also abolished the antiatherogenic effect of wogonin in vivo (Figure 7G,H). In addition, KLF11 knockout abolished the inhibitory effect of wogonin on foam cell formation and inflammation (Figure 7I; and Figures S11 and S12A, Supporting Information), whereas overexpression of KLF11 enhanced the inhibitory effect of wogonin on inflammation (Figure S12B, Supporting Information). More importantly, KLF11 knockout promoted glycolysis and abolished the inhibitory effect of wogonin on glycolysis (Figure 7J–M), whereas overexpression of KLF11 reduced the glycolysis (Figure S13A–D, Supporting Information). PFKFB3 is a key gene that is responsible for glycolysis and the downstream gene of YAP1.^[^ ^42^ ^]^ Notably, neither genetic ablation nor overexpression of KLF11 altered PPARα‐CPT1α signaling activity, yet significantly modulated the key glycolytic enzyme PFKFB3 expression (Figure S13E,F, Supporting Information), which indicated that KLF11 acts as a suppressor in YAP1‐mediated glycolysis. These data may account for the invalid inhibitory effect of wogonin on glycolysis and atherogenesis in KLF11^−/−^ macrophages or mice. Taken together, KLF11 can recruit PPARα and YAP1 to form a transcription complex, in which KLF11 acts as a brake in YAP1‐mediated glycolysis without affecting the PPARα‐CPT1α signaling pathway. Wogonin reprogrammed the metabolism by promoting the interaction between PPARα‐KLF11‐YAP1, by which wogonin reduced inflammation and foam cell formation and thereby attenuates atherogenesis (Figure 7N).

### 2.8 KLF11 Restoration Attenuated the Atherosclerosis and Enhanced the Plaque Stability via Reducing Glycolysis in KLF11^−/−^ Mice

**Figure 8:**
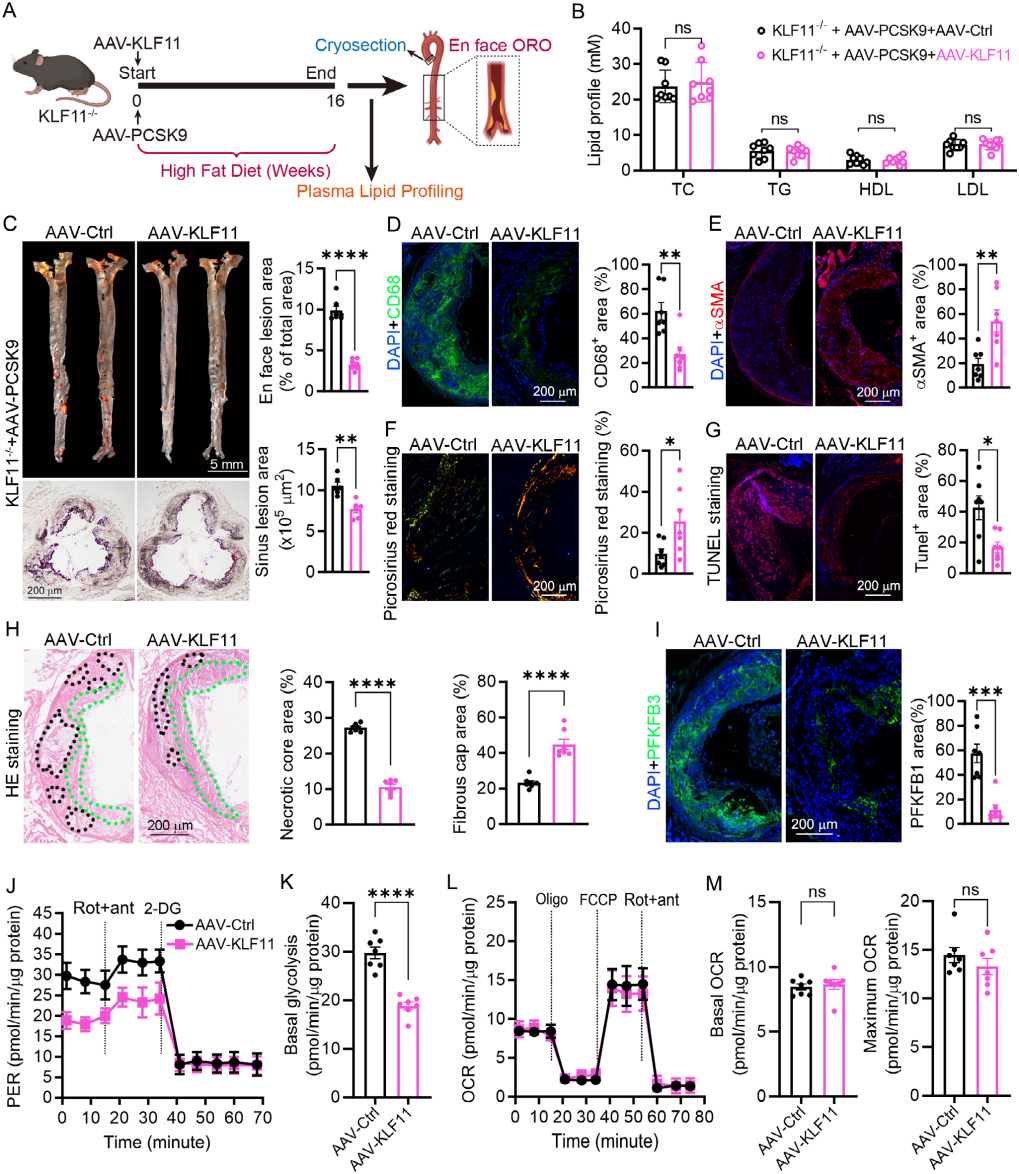
KLF11 restoration attenuated atherosclerosis and plaque vulnerability by reducing glycolysis. A) KLF11^−/−^ mice were infected with AAV‐PCSK9 to induce the atherosclerosis model and simultaneously infected with AAV‐KLF11 to replenish the KLF11. B) The serum profile of KLF11^−/−^ mice infected with AAV‐KLF11, *n* = 7. C) Lesion areas in the whole aorta (Scale bar: 5 mm) and in the aortic root sections (Scale bar: 200 µm) were stained by Oil Red O, *n* = 7. D,E) Immunostaining for CD68 or αSMA and quantification were expressed as the ratio of positive areas to plaque area, *n* = 7. Scale bar: 200 µm. F) Picrosirius red staining for collagen content in plaque, *n* = 7. Scale bar: 200 µm. G) TUNEL staining for cellular apoptosis in the plaque and quantification of positive areas, *n* = 7. Scale bar: 200 µm. H) Hematoxylin and eosin staining was followed by quantitative analysis of the necrotic core area and fibrous cap area in the aortic root. nc: necrotic cores marked by a black dotted line; fc: fibrous cap marked by a blue dotted line, *n* = 7. Scale bar: 200 µm. I) Detection of intraplaque PFKFB1 levels by immunofluorescence staining, *n* = 7. Scale bar: 200 µm. J) PER curves of peritoneal macrophages from KLF11^−/−^ mice infected with AAV‐Ctrl or AAV‐KLF11, *n* = 7. K) Tracking and quantifying the proton efflux rate and basal glycolytic capacity of peritoneal macrophages from KLF11^−/−^ mice infected with AAV‐Ctrl or AAV‐KLF11, *n* = 7, One‐way ANOVA with Tukey's multiple comparisons. L,M) Basal and maximum oxygen consumption were normalized by protein content, *n* = 7, One‐way ANOVA with Tukey's multiple comparisons. Data are presented as mean ± SEM. *p*‐values are shown in the figure B) by two‐tailed *t*‐test or One‐way ANOVA with Tukey's multiple comparisons test C–M). *p < 0.05, **p < 0.01, ***p < 0.001, and ****p < 0.0001 significantly different from the AAV‐Ctrl.

To further determine the protective role of KLF11 in atherosclerosis, the KLF11^−/−^ mice were infected with AAV‐PCSK9 to induce the atherosclerosis model and simultaneously infected with AAV‐KLF11 to replenish the KLF11 (**Figure** **8**A). The result showed that KLF11 restoration did not affect the lipid profile in the serum but mitigated the atherosclerosis and enhanced the plaque stability (Figure 8B–I). In addition, KLF11 replenishment markedly reduced the foam cells in the plaque and decreased the lipid accumulation in the peritoneal macrophages (Figure 8D; and Figure S14A, Supporting Information), suggesting the inhibitory effect on foam cell formation. Furthermore, KLF11 restoration increased the protein level of ABCA1 and ABCG1 in the plaque (Figure S14B,C, Supporting Information). Moreover, KLF11 restoration reduced the inflammation in the mice, which was evidenced by the decreased proinflammatory cytokines and increased anti‐inflammatory cytokines in the plaque, peritoneal macrophages, and serum (Figure S14D–F, Supporting Information). PFKFB3 is a key gene that is responsible for glycolysis and the downstream gene of YAP1.^[^ ^42^ ^]^ KLF11 restoration suppressed expression of PFKFB3 in the plaque (Figure 8J). Moreover, in the peritoneal macrophages from KLF11 restoration mice, the seahorse assay showed that the KLF11 restoration suppressed the glycolysis without affecting the OXPHOS (Figure 8L,M). Collectively, these findings demonstrate that KLF11 reconstitution suppresses glycolysis, thereby attenuating atherosclerotic progression through metabolic reprogramming.

### 2.9 KLF11 Promoted Cholesterol Efflux by Transcriptionally Promoting ABCA1/G1 Expression

**Figure 9:**
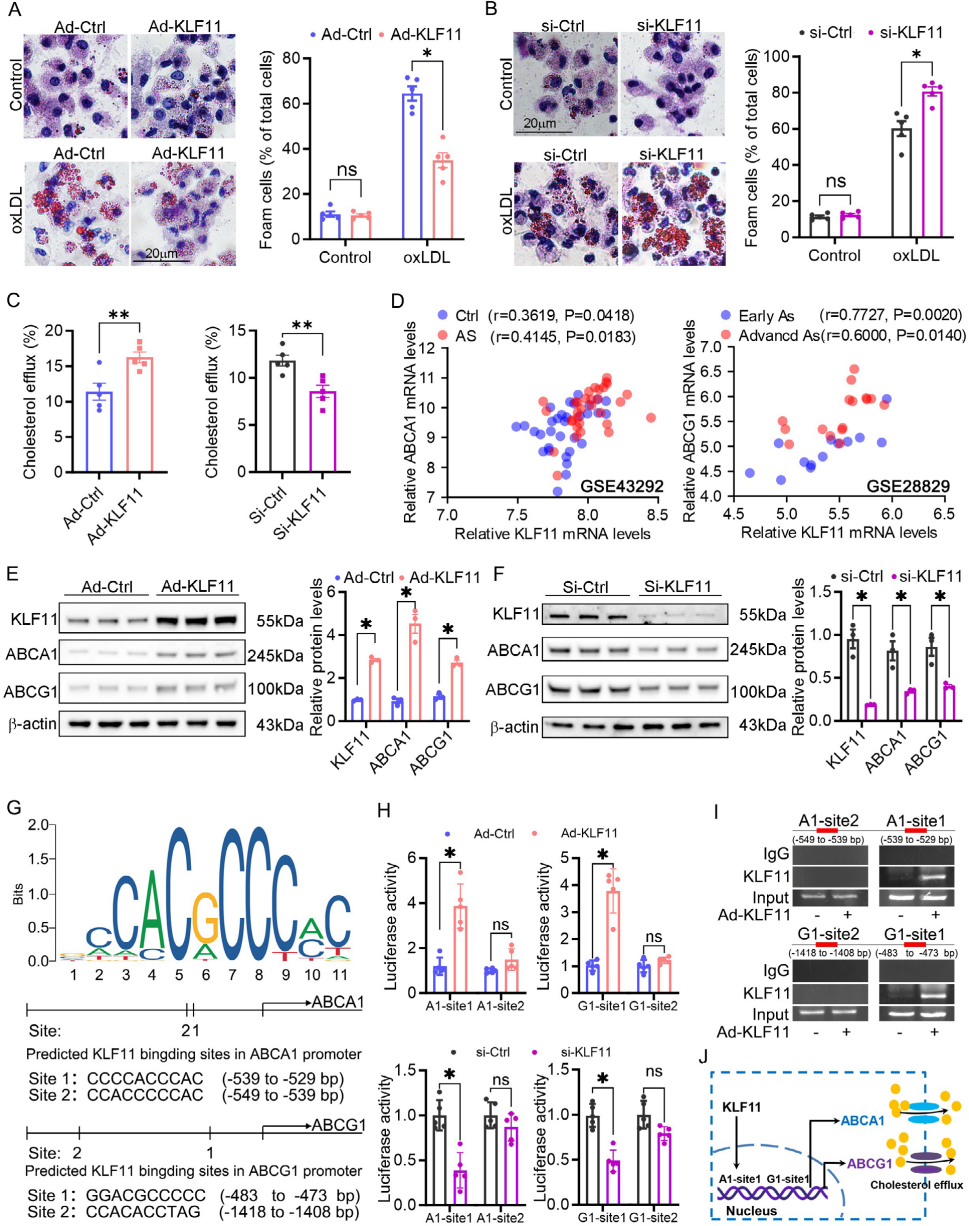
KLF11 promoted ABCA1 and ABCG1 transcription by directly targeting their promoters. A,B) The peritoneal macrophages that were isolated from c57bl6j mice were transfected with Ad‐KLF11 or siKLF11 for 24 h to overexpress or knock down KLF11 before a 24‐h ox‐LDL treatment, followed by the Oil red O staining, *n* = 5. Scale bar: 20 µm. C) The peritoneal macrophages that were isolated from c57bl6j mice were transfected with Ad‐KLF11 or siKLF11 to overexpress or knock down KLF11 and then evaluated the level of cholesterol efflux, *n* = 5. D) KLF11, ABCA1, and ABCG1 expression in human atherosclerotic plaque were obtained from Gene Expression Omnibus databases (GSE28829, GSE43292). Spearman correlation analysis of the transcription levels of KLF11, ABCA1, and ABCG1 showed that KLF11 mRNA levels positively correlate with ABCA1 and ABCG1 levels. E,F) The peritoneal macrophages that were isolated from c57bl6j mice were transfected with Ad‐KLF11 or siKLF11 to overexpress or knock down KLF11 and then evaluated the level of KLF11, ABCA1, and ABCG1, *n* = 5. G) Predicted KLF11 sequence logo and the binding sites of KLF11 at ABCA1 and ABCG1 promoter by JASPAR database (http://jaspar.genereg.net/). H) Luciferase activity of the ABCA1 and ABCG1 promoter in peritoneal macrophages infected with Ad‐KLF11 or siKLF11 for 24 h, *n* = 5. I) DNA fragments that contain the flanking region of the KLF11 on the ABCA1 and ABCG1 promoter were immunoprecipitated with the anti‐KLF11 in macrophages. Chromatin immunoprecipitation (ChIP) analysis of KLF11 binding activity at the ABCA1 and ABCG1 promoter. J) Schematic of the role of KLF11 as a positive regulator of ABCA1 and ABCG1 expression. Data are presented as mean ± SEM. *p*‐values are shown in the figure A,B,C,E,F,H) by Unpaired *t*‐test. *p < 0.05, **p < 0.01, and ***p < 0.001, significantly different as indicated; ns: not significantly different.

To further validate the role of KLF11 in foam cell formation, RAW264.7 cells were transfected with si‐KLF11 or Ad‐KLF11 to knockdown or overexpress KLF11 respectively. The data showed that KLF11 overexpression markedly blocked the foam cell formation, whereas KLF1 knockdown promoted the foam cell formation (**Figure** **9**A,B). Accordingly, KLF11 overexpression promoted cholesterol efflux, which is conversely to that in the KLF11‐knockdown cells (Figure 9C). Meanwhile, we found that KLF11 overexpression did not affect the cholesterol uptake and the expression of scavenger receptors (Figure S15A,B, Supporting Information). These data suggest that KLF11‐induced cholesterol efflux may be ABCA1/G1‐dependent. In this study, we found that wogonin not only promoted ABCA1 and ABCG1 expression but also biased the metabolic mode of macrophage from glycolysis toward OXPHOS, which synergistically promoted cholesterol efflux (Figures 3 and 6). Therefore, enhanced energy supply and transporter support by wogonin can contribute to the promoted cholesterol efflux and inhibition of foam cell formation. However, whether the promoted expression of ABCA1 and ABCG1 was associated with KLF11 remains unknown. Correlation analysis revealed that the KLF11 levels in the aorta were positively correlated with the ABCA1 and ABCG1 levels in normal controls and carotid atherosclerosis patients (Figure 9D), suggesting that KLF11 regulates ABCA1 and ABCG1 expression. Indeed, KLF11 overexpression led to ABCA1/G1 expression, whereas, KLF11 knockdown resulted in decreased ABCA1/G1 expression (Figure 9E,F). Furthermore, we searched for potential KLF11‐binding sites in the promoters of ABCA1 and ABCG1 (within the −2.0 kb fragment upstream of the transcription start site) by using the JASPAR database. Consequently, four potential sites were predicted in each promoter region (Figure 9G). Luciferase assay showed that KLF11 overexpression or knockdown stimulated or inactivated ABCA1/G1 transcriptional activity by binding ABCA1‐site1 and ABCG1‐site1 (Figure 9H). ChIP‐qPCR revealed that the KLF11 enrichment on sites of the ABCA1 and ABCG1 promoter fragments was enhanced by Ad‐KLF11 overexpression (Figure 9I). Collectively, these results suggest that KLF11 promotes ABCA1 and ABCG1 transcription by targeting their promoters, which supports cholesterol efflux.

## 3 Discussion

Atherosclerosis, a major cause of cardiovascular disease, is a chronic inflammatory disease caused by the accumulation of lipid‐loaden foam cells/macrophages in the walls of the arteries.^[^ ^43^ ^]^ Metabolic condition of macrophages determines lipid efflux and inflammatory stage during atherogenesis.^[^ ^44^ ^]^ Notably, macrophage‐rich atherosclerotic arteries are highly active in glycolysis, inhibition of which reduces atherogenesis in preclinical models.^[^ ^45^ ^]^ Therefore, metabolic reprogramming of macrophages is a novel strategy to treat atherosclerosis. In this study, wogonin significantly reduced the lesion area and altered the lipid profile in LDLR‐deficient mice, implying that wogonin displays an antiatherogenic effect.

Vulnerable plaques prone to rupture are a principal contributor to adverse cardiovascular events.^[^ ^46^ ^]^ Noticeably, as a dominant source of chemokines, cytokines, and matrix protein‐degrading enzymes, macrophages have a critical function in the local inflammatory response and plaque rupture.^[^ ^47^ ^]^ Our results suggest that wogonin reduced macrophage accumulation. In addition, the pathology of vulnerable atherosclerotic plaques is characterized by thinning of the fibrous cap, structural instability, and enlargement of the necrotic core, which are mainly caused by MMP and cathepsin‐mediated degradation of the ECM components, especially collagen and elastin.^[^ ^48^ ^]^ In the microenvironment of inflamed plaques, the accumulation of apoptotic cells acts cooperatively with chronic inflammation to amplify MMP and cathepsin secretion and activity, thereby exacerbating plaque instability and leading to plaque rupture.^[^ ^49^ ^]^ Previous studies have identified wogonin as a suppressor of MMP‐9 expression.^[^ ^50^ ^]^ Our findings corroborate these observations, demonstrating that wogonin treatment significantly reduces MMP‐2/9 and cathepins S/K levels within plaques, which enhances fibrous cap thickness by mitigating collagen and elastin degradation. These effects collectively indicate wogonin's capacity to stabilize plaques through ECM preservation. Notably, advanced plaques exhibit heightened VSMCs apoptosis, a phenomenon potentially linked to inflammatory macrophage activity that suppresses collagen synthesis and aggravates fibrous cap thinning.^[^ ^51^ ^]^ According to our results, wogonin inhibit VSMCs apoptosis. We speculate that the protective effect may partly derive from improving the macrophage inflammatory response and blocking the inflammatory cascade of plaque development.

The imbalance between proinflammatory and anti‐inflammatory processes contributes to chronic inflammation and the formation of atherosclerotic plaques.^[^ ^52^ ^]^ Targeting inflammation could provide a promising avenue to prevent and treat atherosclerosis.^[^ ^53^ ^]^ Activation of PPARα can reduce inflammation to attenuate atherosclerosis.^[^ ^54^ ^]^ In this study, wogonin reduced the level of systemic inflammation in LDLR^−/−^ mice and promoted macrophage polarization from the M1 to M2 type, which contributed to the antiatherogenic effect of wogonin. Moreover, preclinical studies in mice have demonstrated that Ly6C^high^ monocytes contribute to both the growing atheroma and plaque instability.^[^ ^55^ ^]^ Noticeably, wogonin induced differentiation of circulating monocytes to the Ly6C^low^CX3CR1^high^ subtype, which further contributed to the inhibitory effect of wogonin on atherogenesis.

Moreover, wogonin promotes cholesterol efflux by upregulating levels of the ABCA1 and ABCG1 to reduce the lipid load in macrophages and the following foam cell formation. RNA‐seq revealed activation of the PPARα pathway in wogonin‐treated macrophages, suggesting that wogonin may be a natural PPARα agonists, which was also confirmed by other wogonin related pharmacological studies.^[^ ^56^ ^]^ The previous study showed that activation of PPARα induces the expression of ABCA1, which promotes cholesterol efflux from macrophages.^[^ ^57^ ^]^ Accordingly, activation of PPARα signaling by wogonin partially accounts for the increased ABCA1 expression and cholesterol efflux. Noticeably, we found that PPARα can recruit KLF11 and YAP1 to form a transcription complex. Moreover, KLF11 levels in the aorta were positively correlated with the ABCA1 and ABCG1 levels in normal controls and carotid atherosclerosis patients. Furthermore, we found that KLF11 can promote the expression of ABCA1 and ABCG1, which suggested that KLF11 acts as a positive regulator of ABCA1 and ABCG1 expression and cholesterol efflux, and inhibits the foam cell formation. In addition, ABCA1‐mediated cholesterol efflux from macrophages is regulated by mitochondrial ATP production.^[^ ^15^ ^]^ In our study, wogonin promoted the FAO to produce more ATP, which indirectly supported the cholesterol efflux from the macrophage. PPARα agonists such as fibrates are widely prescribed for the treatment of dyslipidemias as lipid‐lowering drugs in the clinic.^[^ ^58^ ^]^ In our study, wogonin improved the lipid profile, which may be dependent on its stimulation of the PPARα signaling pathway. Augmenting mitochondrial‐dependent FAO potentially slows atherosclerotic progression.^[^ ^31^ ^]^

PPARα‐CPT1α axis regulates the process of lipid metabolism.^[^ ^59^ ^]^ Wogonin promoted PPARα nuclear translocation to facilitate CPT1α transcription, which promoted the FAO. In contrast, inhibition of CPT1α or PPARα leads to disruption of FAO, which resulted in a more severe inflammatory state and lipid accumulation as well as atherogenesis, and abrogated the antiatherogenic effects of wogonin, indicating that PPARα‐CPT1α is an important pathway for the efficacy of wogonin. Metabolic reprogramming of macrophages regulates inflammation. M1 macrophages metabolize mainly by glycolysis to meet the need for a rapid energy supply. In contrast, M2 macrophages prefer to increase FAO to support OXPHOS to produce ATP consistently and steadily.^[^ ^6^ ^]^ The inflammatory phenotype of macrophages can determine their metabolic phenotype and vice versa.^[^ ^10^, ^60^ ^]^ Our findings suggest that wogonin profoundly reshaped the metabolism of macrophages in LDLR^−/−^ mice, promoting OXPHOS while decreasing the level of glycolysis and increasing the intracellular ATP content. In parallel, wogonin‐mediated metabolic remodeling also reprogrammed the ratio of macrophage subpopulations, with an upregulation of the M2 macrophage ratio to deliver anti‐inflammatory effects. In addition, PPARα inhibition leads to the disruption of intracellular OXPHOS accompanied by increased levels of glycolysis. More importantly, the PPARα inhibitor GW6471 inverted the effect of wogonin on metabolic reprogramming in macrophages and, consequently, reversed the antiatherogenic efficacy, suggesting that the protective effects of wogonin were mediated by PPARα. The structural and functional integrity of the mitochondria is necessary for OXPHOS and FAO.^[^ ^61^ ^]^ However, in pathological conditions of atherosclerosis, ox‐LDL mediates LCFA transport to mitochondria but inhibits FAO, leading to LCFA accumulation, damage in mitochondria, and the production of ROS.^[^ ^62^ ^]^ Mitochondria ROS (mitoROS) signaling plays a major role in atherosclerosis. Suppression of mitoROS signaling in macrophages reduces lesion area, inflammatory signaling, and immune cell infiltration into the aortic root of LDLR^−/−^ mice.^[^ ^63^ ^]^ A previous study showed that wogonin targets mitochondria to exert its pharmacological effect.^[^ ^64^ ^]^ Our study found that wogonin significantly reduced ROS levels in ox‐LDL‐stimulated cells and protected mitochondrial structures from ox‐LDL‐induced damage. PPARα deficiency led to mitochondrial dysfunction,^[^ ^65^ ^]^ which accounts for the protective effect of wogonin on mitochondrial function and structure. Furthermore, wogonin reduces oxidative stress and promotes mitochondrial biogenesis to facilitate mitochondrial repair, and these pharmacological effects are mediated in a PPARα‐dependent manner.

In this study, RNA‐seq suggested that wogonin promoted the expression of KLF11. The structural diversity of N‐terminal amino acid residues in the KLF family enables KLF members to recruit specific corepressors or coactivators, and thus play different transcriptional regulatory roles depending on the binding environment.^[^ ^66^ ^]^ Previous studies have demonstrated that KLF11 suppresses NF‐κB transcriptional activity through direct interaction with p65,^[^ ^67^ ^]^ concurrently inhibiting MMP9 transcription via dual mechanisms: NF‐κB pathway‐dependent regulation and direct binding to the MMP9 promoter.^[^ ^68^ ^]^ Notably, our results and previous reports also elucidate the inhibitory effect of wogonin on MMP9 expression,^[^ ^50^, ^69^ ^]^ which may be mediated in part through KLF11. Furthermore, KLF11 enhances PPARα expression via a nontranscriptional regulatory mechanism.^[^ ^70^ ^]^ Intriguingly, KLF11 has been identified as a putative PPARα target gene, suggesting the existence of a bidirectional regulatory circuit between these factors.^[^ ^71^ ^]^ This reciprocal interaction likely constitutes a positive feedback loop, highlighting the pivotal role of KLF11 in orchestrating metabolic homeostasis through integrated transcriptional and post‐transcriptional mechanisms. YAP1 recently has been described as a key metabolic hub in the regulation of glycolysis.^[^ ^72^ ^]^ YAP1 can promote glycolysis and binding of YAP1 with the PPARα can induce nuclear translocation of YAP1.^[^ ^40^ ^]^ KLF11 can suppress the YAP1 signaling pathway by recruiting YAP1.^[^ ^41^ ^]^ These studies indicated that KLF11 may act as a brake in the PPARα‐YAP1 signaling pathway, which plays a critical role in the antiatherogenic effect of wogonin via regulating metabolic metabolism. In this study, wogonin promoted the expression of KLF11, by which wogonin suppressed YAP1‐mediated glycolysis and exerted the antiatherogenic effect. However, KLF11 knockout not only exacerbated atherosclerosis but also markedly abolished the antiatherogenic effect of wogonin by promoting glycolysis. In contrast, KLF11 gain‐of‐function attenuates atherosclerosis and enhances plaque stability via reducing glycolysis in KLF11^−/−^ mice. These data suggested that KLF11 acts as a suppressor of atherogenesis and glycolysis. Mechanistically, we found that under wogonin stimulation, PPARα can recruit KLF11 and YAP1 to form a transcription complex, in which KLF11 suppressed the YAP1‐mediated glycolysis but did not affect the PPARα‐CPT1α‐mediated FAO and mitochondrial protection.

In conclusion, this study indicated that KLF11 can recruit PPARα and YAP1 to form a transcription complex, in which KLF11 acts as a brake in PPARα‐YAP1‐mediated glycolysis. KLF11 acts as a positive regulator on ABCA1/G1‐mediated cholesterol efflux thereby inhibiting foam cell formation. Wogonin reshapes the metabolism of macrophages from glycolysis to FAO and promotes OXPHOS dependently on the PPARα‐KLF11‐YAP1 pathway, by which wogonin reduces the inflammation and foam cell formation and thereby attenuates atherogenesis. A notable limitation hindering the therapeutic application of wogonin is the low oral bioavailability.^[^ ^73^ ^]^ Therefore, emerging pharmaceutical strategies involving nanocapsule technology or targeted chemical structure modification have considerable potential to improve the bioavailability and pharmacokinetic profile of wogonin.^[^ ^74^ ^]^ Our study suggested that KLF11 may be a new antiatherogenic target and wogonin may be a novel strategy for atherosclerosis treatment.

## 4 Experimental Section

### Reagents

Rabbit anti‐ABCG1 (Cat#: NB400‐132) and ABCA1 (Cat#: NB400‐105) polyclonal antibodies were purchased from Novus Biologicals (Littleton, CO). Mouse anti‐IL‐1β (Cat#:12242S) monoclonal antibody was purchased from Cell Signaling Technology (Danvers, MA). Mouse anti‐Arg (Cat#: ab239731) and KLF11 (Cat#: ab279389) monoclonal antibody was purchased from Abcam (Cambridge, MA). Rabbit anti‐CD68 (Cat#: ab283654), Lamin A/C (Cat#: ab169532), and YAP1 (ab52771) monoclonal antibodies were purchased from Abcam (Cambridge, MA). Mouse anti‐MMP2 (Cat#:sc13594), αSMA (Cat#:sc130617), PPARα (Cat#: sc398394), CPT1α (Cat#: sc393070), and CD68 (Cat#: sc20060) monoclonal antibodies were purchased from Santa Cruz Biotechnology, Inc (Santa Cruz, CA). Rabbit anti‐Beta Actin (Cat#: BM3873) polyclonal antibody was purchased from Boster Biological Technology Co., Ltd (Wuhan, China). Rabbit anti‐ACADL (Cat#: 17526), anti‐FABP2 (Cat#: 21252), and anticleaved‐caspase3 (Cat#: 25128) polyclonal antibodies were purchased from Proteintech Group, Inc (Wuhan, China). Rabbit anticathepsin S (Cat#: bs8558R) and anticathepsin R (Cat#: bs1611R) polyclonal antibodies were purchased from Biosynthesis Biotechnology CO., LTD (Beijing, China). Mouse FTTC‐anti‐αSMA (Cat#: F3777) was purchased from Sigma‐Aldrich CO., LTD. (St. Louis, USA). FITC antimouse CD3 antibody (Cat#: 100203) was purchased from Biolegend, Inc. (San Diego, USA). Mouse antirabbit IgG‐R (Cat#: sc2492), mouse antirabbit IgG‐FITC (Cat#: sc2359), m‐IgGκ BP‐PE (Cat#: sc516141), and m‐IgGκ BP‐FITC (Cat#: sc516140) antibodies were purchased from Santa Cruz Biotechnology, Inc (Santa Cruz, CA). TG (Cat#: 100020090), CHO (Cat#: 100020080), LDL‐C (Cat#: 100020245), HDL‐C (Cat#: 100020235), AST (Cat#: 100020010), and ALT (Cat#: 100020080) kit were purchased from Biosino Bio‐Technology and Science, Inc (Beijing, China). GW6471 (Cat#: S2798) was purchased from Selleck Biotechnology Co., Ltd (Selleck, TX). Wogonin (Cat#: 632‐85‐9) was purchased from Shanghai Yuanye Bio‐Technology Co., Ltd (Shanghai, China). AAV‐PCSK9, (CAT# GOSV0335434_1) was purchased from Shanghai Genechem Co., Ltd.

### Atherosclerotic Models were Induced in LDLR^−/−^ and KLF11^−/−^ Mice

The protocol for in vivo studies was approved by the Ethics Committee of Tianjin University of Traditional Chinese Medicine and conform to the Guide for the Care and Use of Laboratory Animals published by the NIH (NIH publication, eighth edition, updated 2011). This study obtained approval from the Ethics Committee (Approval No. DXBYY‐IACUC‐2020, 10/11/2020) and Laboratory Animal Use License No. (SYXK(Jin)2020‐0001). Eight‐week‐old, male LDLR^−/−^ mice were purchased from Changzhou Kavins Laboratory Animal Co.Ltd (Nanjing, Jiangsu, China). KLF11^−/−^ mice were purchased from Gempharmatech Co. Ltd (Jiangsu, Nanjing, China), and subsequently backcrossed with C57BL/6 mice to ensure genetic stability and compatibility. The production of knockout mice with a homozygous (−/−) genotype and their wild‐type littermates with a homozygous (+/+) genotype was accomplished through the strategic intercrossing of heterozygous mice bearing a (±) genotype. Specifically, male mice at the age of 8 weeks were selected as experimental subjects for identification of genotypes. The genotyping of the KLF11^−/−^ mice was confirmed through agarose gel electrophoresis of the PCR fragments in the CRISPR‐targeting region amplified from genomic DNA isolated from mouse tails with the following primers: Forward 5’‐CTCTCACCTGACCTCCTCCCT‐3’ and Reverse 5’‐ AGTCCCCATTCTCTCCTCCCT‐3’. The primer sequence for the other band is: Forward 5’‐CCCAGAACACTGAGCAAGAGG‐3’ and Reverse 5’‐ AAGCAAGGCTGACAGGAAACC‐3’. The mice were housed in a temperature‐controlled room with a 12‐h light/12‐h dark cycle under pathogen‐free conditions and had free access to food and water. The dosing regimen for wogonin was determined based on prior pharmacological studies.^[^ ^18^, ^75^ ^]^ The LDLR^−/−^ mice subjected to a model of atherosclerosis were randomly divided into three groups: HFD (21% fat plus 0.5% cholesterol, MD12015HL, Medicience Ltd., China) group, HFD containing low dose Wog [Wog‐L, 10 mg day^−1^ kg^−1^body weight (mpk)] and high dose Wog (Wog‐H,25 mpk). After treatment for 16 weeks, the mice were euthanized and their aortas, peritoneal macrophages, livers, and blood samples were collected as previously reported. In the same setting, LDLR^−/−^ mice on high‐fat chow were fed the PPARα inhibitor GW6471 (20 mpk) or CPT1α inhibitor etomoxir (20 mpk) in the presence or absence of wogonin to further explore whether the antiatherogenic effect of wogonin was associated PPARα or CPT1α activation. To determine the role of KLF11 in metabolic reprogramming and atherosclerosis development, 8‐week‐aged KLF11^−/−^ and WT mice were fed with a 16‐week HFD and simultaneously given AAV‐PCSK9 (200µL, 3×10^11^ vg/mice) through tail vein injection to construct atherosclerosis model. After being treated for 16 weeks, all mice were euthanized by cervical dislocation after an overdose of isoflurane, followed by collection of aorta, liver, peritoneal macrophage, and blood samples.

### Determination of Serum Lipid Profile and Liver Function in Atherosclerotic Mice

The blood was rested for 4 h and centrifuged (3000 rpm) to obtain the serum. Subsequently, the serum was used to test for total cholesterol (Total‐C, TC), high‐density lipoprotein (HDL)‐C, low‐density lipoprotein (LDL)‐C, triglycerides (TG), AST, and alanine aminotransferase (ALT) by the fully automatic biochemical analyzer.

### High Throughput Assays for Serum Inflammatory Factors

Several inflammatory factors in the serum were detected by the LEGENDplexTM Mouse Anti‐Virus Response Panel (Cat. No. 740622, Biolegend). The reagent containing the microspheres was shaken for 1 min to avoid precipitation of the microspheres. Subsequently, the reagents containing the microspheres were added to a “V‐bottom” 96‐well plate. Gradient‐diluted standards and twofold diluted serums were added to the well of the 96‐well plate described above. The systems were incubated on a shaker oscillator for 2 h at 800 rpm and room temperature, in the dark. The tubes were washed and resuspended, 25 µL of premixed antibody was added to each well and then incubated for 1 h at 800 rpm and sheltered on a shaker. After two washes, 150 µL of Assay Buffer was added to each well to resuspend all microspheres. Two gates were created to obtain Beads A and B when the X‐axis was FSC, and the Y‐axis was SSC in the flow cytometer. The voltage was adjusted to ensure that both groups of Beads A and B microspheres fell in the gate when the X‐axis was PE and the Y‐axis was APC. The settings were saved, and the samples were run in the flow cytometer.

### Atherosclerotic Lesion Analysis

The aorta, including the ascending arch, thoracic, and abdominal segments, and aortic root were isolated and gently cleaned of the adventitia. To assess the area of the lesion, the whole aorta was collected and stained with Oil Red O solution. To quantify plaque burden, paraformaldehyde‐fixed aortic roots were placed in an optimal cutting medium and sectioned.^[^ ^76^ ^]^ Lipid deposition, necrotic core, fibrous cap, collagen content, apoptosis rate, and expression of CD68, CD3, αSMA, MMP2/9, cleaved‐Caspase 3, and cathepsin S/K protein in lesion areas were determined by Oil Red O staining, Hematoxylin and eosin (H&E), Elastic Van Gieson (EVG), TUNEL staining and immunofluorescent staining with aortic root cross sections, respectively. In addition, coexpression regions of CD68 with Arg1, IL‐1β, ABCA1, ABCG1, CPT1α, and PPARα in the plaques were also presented by immunofluorescence staining. All the images were obtained with a microscope and quantified lesion areas in en face aorta and aortic root cross sections, respectively, using a computer‐assisted image analysis protocol (Photoshop CS6 and Image J).

### Cell Lines and Culture Conditions

All cell lines were purchased from ATCC (Manassas, VA). RAW264.7 cells and peritoneal macrophages were cultured in a complete 1640 medium containing 10% FBS, 50 µg mL^−1^ penicillin/streptomycin, and 2 mm glutamine. The method of isolation of peritoneal macrophages from mice has been described previously.^[^ ^77^ ^]^ After the mice were anesthetized and euthanized, 10 mL of PBS was injected into the abdomen.^[^ ^78^ ^]^ The supernatant was discarded after the liquid was pumped out and centrifuged (1000 rpm, 5 min). The cells at the bottom were resuspended in 1640 medium and plated in 6‐well plates for the following assay.

### MTT Assay

RAW264.7 cells were inoculated in 96‐well plates and incubated for 16 h in a 37 °C incubator to adhere the cells to the culture dish. Then, the cells were incubated with different concentrations of wogonin. After 24 h, the drug‐containing medium was replaced by an MTT‐containing medium and the cells were cultured for 4 h. Finally, 100 µL of formazan lysate was added to each well and the cells were incubated for 4 h until the purple crystals could not be observed under the microscope. The absorbance at 570 nm was measured using a microplate reader.

### Determination of Foam Cell Formation In Vitro and In Vivo

In vitro, RAW264.7 cells were seeded on coverslips in 24‐well plates. After attachment, RAW264.7 cells were incubated with a serum‐free 1640 medium containing 100 µg mL^−1^ of ox‐LDL for 3 h and then incubated for a further 16 h with or without wogonin in the medium. Cells were fixed by paraformaldehyde and then stained with Oil Red O solution.^[^ ^79^ ^]^ In vivo, mice peritoneal macrophages were isolated and planted on slides in 24‐well plates. Peritoneal macrophages that adhere to the culture dish were fixed with paraformaldehyde and stained with Oil Red O solution. As previously reported,^[^ ^77^ ^]^ cells containing lipid droplets (>10/cell) were considered foam cells, and >10 fields/sample were counted.

### Determination of Monocyte and Macrophage Polarization by Flow Cytometry

Monocyte subtypes in the whole blood of mice were detected by flow cytometry. Peripheral blood monocytes were blocked from nonspecific binding by Fcγ block (Cat: 101319, Biolegend). Monocytes were delineated by being stained with APC‐labeled anti‐CD11b (Cat: 128005, Biolegend). Different monocyte subtypes were marked by FITC‐labeled anti‐CX3CR1 (Cat: 149019, Biolegend) and PE‐labeled anti‐Ly6C (Cat: 128007, Biolegend). After staining, the erythrocytes in the whole blood were lysed. Monocytes were differentiated into the Ly6C^high^CX3CR1^low^ population and Ly6C^low^CX3CR1^high^ population by different gating using flow cytometry.

Peritoneal macrophages were extracted from mice of Ctrl, Wog, GW6471, and GW6471+Wog groups. Peritoneal macrophages were stained with FITC‐labeled anti‐F4/80 (Cat: 157309, Biolegend) and PreCP‐labeled anti‐CD11b (Cat: 101229, Biolegend) for identification. PE‐labeled anti‐CD86 (Cat: 159203, Biolegend) and APC‐labeled anti‐CD11c (Cat: 117309, Biolegend) were used to mark M1 macrophages, while PE‐labeled anti‐CD206 (Cat: 141705, Biolegend) and APC‐labeled anti‐CD163 (Cat: 155305, Biolegend) were used to identify M2 macrophages. Meanwhile, RAW264.7 cells treated with GW6471 for 48 h and/or wogonin for 16h were stained with PE‐labeled anti‐CD206 (Cat: 141705, Biolegend) and FITC‐labeled anti‐CD86 (Cat: 561962, BD Pharmingen). The proportion of each subpopulation was examined to determine the effect of wogonin on macrophage polarization.

### Cholesterol Efflux and Uptake Assays

RAW264.7 cells were grown in 24‐well plates and incubated with 3‐dodecanoyl‐NBD cholesterol (1 µg mL^−1^, Cayman Chemical) for 6 h after the cells adhered to the culture dish. Post incubation, the medium was removed, and the cells were washed three times with PBS. Cells were added to a serum‐free medium containing Apo AI (5 µg mL^−1^) and HDL (20 µg mL^−1^) as cholesterol receptors and incubated with or without wogonin for 6 h. Finally, cell supernatants were collected, and cells were washed with PBS and then lysed with 100 µL lysis solution (Cat: 1067‐100, Biovision). Finally, the supernatant and cell lysate from each well were transferred to two white 96‐well plates (with opaque flat bottom wells) and fluorescence (*E*_x_/*E*_m_ = 485/523 nm) was measured in endpoint mode. The cholesterol efflux from labeled macrophages was calculated by dividing the fluorescence intensity obtained from the supernatant by the sum of the fluorescence intensity of the supernatant and cell lysate from the same treatment.

ox‐LDL was labeled with l, l’‐dioctadecyl‐3,3,3’,3’‐tetramethyl‐indocarbocyanine perchlorate (DiI‐ox‐LDL, Invitrogen), as previously described. The cells were transfected with Ad‐KLF11 and then incubated with Dil‐ox‐LDL (10 µg mL^−1^) for 2 h at 4 °C or 4 h at 37 °C. They were then fixed with 4% paraformaldehyde for 10 min, followed by the staining of the nuclei with DAPI. After washing with PBS, the cells were photographed using a microscope, and the fluorescence intensity was quantified using ImageJ software.

### Determination of Target of Wogonin by RNA‐seq Analysis

Total RNA from cells was carefully extracted by Trizol reagent (CAT: 15596026, Thermo Fisher), and RNA quality was assessed by an Agilent 2100 Bioanalyzer (Agilent Technologies, Palo Alto, CA). The mRNA was enriched by Oligo (dT) beads and reverse transcribed into cDNA by random primers. The cDNA fragments were then purified using the QiaQuick PCR extraction kit (Qiagen, Venlo, The Netherlands), end‐repaired, PolyA added and ligated to the Illumina sequencing junction. The ligated products were screened by agarose gel electrophoresis, amplified by PCR, and sequenced using Illumina HiSeq2500 (Gene Denovo Biotechnology, Guangzhou, China). Differential RNA expression between the two groups was analyzed using DESeq. Genes/transcripts with false discovery rate (FDR) parameters below 0.05 and absolute fold change ≥ 2 were considered differentially expressed genes. Gene ontology (GO) terms and Kyoto Encyclopedia of Genes and Genomes (KEGG) pathways were enriched in differentially expressed genes in macrophages after wogonin treatment.

### Mitochondrial Copy Number Determination

Total DNA was carefully extracted using the ScienCell SpeeDNA Isolation Kit (Cat: MB6918) and the procedure was carried out strictly according to the instructions. Mitochondrial DNA was amplified using the Relative Mouse Mitochondrial DNA Copy Number Quantification qPCR Assay Kit (Cat: M8938). Each sample was taken in a total system of 20 µL and the amplification program was set according to the instructions. The fold of the administered group versus the control group was calculated by 2^−∆Cq (mtDNA) ‐∆Cq (SCR)^.

### Measurements of ROS

RAW264.7 cells were grown in 6‐well plates with or without stimulation and treatment. After a period of 24 h, the drug‐containing medium was discarded, and the cells were washed thrice with PBS. DCFH‐DA (S0033S, Beyotime Biotechnology) was diluted at a ratio of 1:1000 with a serum‐free medium to a final concentration of 10 µm. 1 mL of DCFH‐DA dilution was added to each well and incubated for 20 min at 37 °C in an incubator. Cells were washed three times through a serum‐free cell culture medium to adequately remove DCFH‐DA that had not entered the cells. Finally, RAW264.7 cells were resuspended and were assayed for ROS levels by flow cytometry.

### Evaluation of Metabolic Reprogramming in Macrophages by Seahorse Assay

The extracellular flux of macrophages was monitored in real‐time by assaying OCR and PER with an XF‐96 Extracellular Flux Analyzer (Seahorse Bioscience, North Billerica, MA). Peritoneal macrophages from different groups of mice were inoculated into XF‐96 well plates at a density of 8×10^5^ per well. On the day of the experiment, the medium for the cells was changed from growth medium to assay medium. Before uploading, 1 µm of oligomycin was added sequentially to inhibit ATP synthase; 1.5 εm of FCCP (fluorocarbon cyanide phenylhydrazone) was added to induce mitochondrial uncoupling to determine spare/maximal respiratory capacity and 0.5 µm of rotenone/antimycin A was added to determine nonmitochondrial respiration. Furthermore, in experiments to detect PER, 0.5 µm rotenone/antimycin A was added to interrupt the electron transport chain; 50 mm of 2‐DG was added to inhibit glycolysis (all reagents purchased from Seahorse Bioscience). RAW264.7 cells were treated with wogonin or/and GW6471 for 24 h before the OCR and PER experiments were also performed as described above.^[^ ^80^ ^]^

### Mitochondrial Tracing

RAW264.7 cells were grown in 24‐well plates and incubated with wogonin for 16 h and/or GW6471 for 48 h. After treatment, the drug‐containing medium was discarded, 100 nm MitoLite Green FM staining solution (Cat: 22695, AAT Bioquest) was added and the cells were incubated at 37 °C for 30 min. Cells were washed with 1x Hanks and 20 mm HEPES Buffer and observed through a fluorescent microscope with a FITC filter.

### ATP Measurement in Peritoneal Macrophages and RAW264.7 Cells

Experiments were performed according to the kit protocol (S0027, Beyotime Biotechnology). Lysate (200 µL) was added to each well of the 6‐well plate to lyse the cells. The cell lysate was centrifuged for 5 min at 12 000 × *g* and the supernatant was aspirated to complete subsequent experiments. ATP assay was added to a white 96‐well plate and left at room temperature for 5 min to deplete the substrate ATP. Standard curves were obtained by gradient dilution and 20 µL of sample or standard were added to the ATP assay separately. The chemiluminescence signal was detected by a luminometer and the results were normalized by protein quantification and analyzed statistically.

### Western Blot and Quantitative Real‐Time PCR

Total cellular proteins were extracted from RAW264.7 cells and mice peritoneal macrophages. Protein expression of ABCA1, ABCG1, CPT1α, PPARα, FABP2, ACADL, Lamin A/C, and β‐actin was determined by western blot. Among these, the nucleoproteins were extracted according to the kit instructions (P0027, Beyotime Biotechnology). Total RNA was extracted from cells followed by the determination of mRNA expression by quantitative real‐time PCR (q‐RT‐PCR) with a reverse transcription kit (CAT: R222‐01, Vazyme), an SYBR green PCR master mix (DBI, Bioscience), and the primers with sequences listed in Table S1 (Supporting Information). Expression of IL‐1β, TNF‐α, IL‐6, NFκB, NLRP3, CCL2, CCL5, INF‐γ, iNOS, IL‐12, VCAM, Arg1, TGF‐β, MMP7, MCP‐1, Nrf1, Nrf2, TFAM, SOD2, and HO‐1 mRNA was normalized by β‐actin mRNA in the corresponding samples.

### Molecular Docking

The compound for this docking (Wogonin) was obtained from the PubChem database (https://pubchem.ncbi.nlm.nih.gov/). PPARα (PDB ID: 3ET1) target protein structures were obtained from the RCSB database (https://www.rcsb.org/). Protein structures were processed on the Maestro 11.9 platform, where proteins were removed from crystalline water, missing peptides were repaired, and finally, proteins were optimized for energy minimization, as well as geometry, by Schrodinger's Protein Preparation Wizard.^[^ ^81^ ^]^ The processing and optimization of the virtual screening were performed by the Glide module in the Schrödinger Maestro software. The receptors were preprocessed, optimized, and minimized (constrained minimization using the OPLS3e force field). All compounds were prepared according to the default settings of the LigPre module. The original ligand of the protein was selected as the center of mass for the 10 Å box. The interaction with the active site was obtained by analyzing the mode of action of the compound and the target protein, such as the resulting hydrogen bonding, π–π interactions, and hydrophobic interactions.

### Surface Plasmon Resonance

The surface plasmon resonance analysis was performed on a Biacore 8K+ instrument (cytiva, USA) according to the protocol of the manufacturer. Recombinant mouse PPARα protein (12080‐H07E, Sino Biological, Inc., Guangzhou, China) was immobilized on a CM7 chip. At concentrations ranging from 1.5625–50 εm, wogonin was used. The KD was calculated using Biacore T200 evaluation software after running a steady‐state affinity model.

### Immunoprecipitation Assay

Immunoprecipitation assay was performed as described.^[^ ^82^ ^]^ Cell lysates were prepared by incubation with lysis buffer supplemented with protease inhibitor cocktails for 30 min at 4 °C, which was followed by centrifugation at 12 000 rpm for 15 min at 4 °C. Control or specific antibodies (anti‐KLF11, anti‐PPARα, anti‐YAP1, anti‐IgG) were added into cell lysates for 12 h at 4 °C with constant rotation; 30 µL of prewashed protein A/G agarose beads were added and incubated for an additional 2 h. Magnetic beads were added to cell lysates and gently rotated at 4 °C for 12 h. The precipitated proteins were eluted from the beads by resuspending the beads in 2xSDS‐PAGE loading buffer and boiling for 5 min, which were subjected to SDS‐PAGE, followed by immunoblotting with appropriate antibodies.

### Data and Statistical Analysis

All statistical metrics included data from at least three independent samples. Values (control and test groups) were normalized to the mean of the experimental controls. The data are presented as the means ± SEMs. Normality was assessed by D'Agostino–Pearson (*n* ≥10) and Shapiro–Wilk test (*n* < 10). Data homoscedasticity was examined using the Bartlett and Brown–Forsythe test. Differences between two groups were determined by unpaired Student's *t*‐test; between multiple groups were determined by one‐way ANOVA and Dunnett's or Tukey's HSD post hoc tests. For multiple comparisons, the Kruskal–Wallis test was used for non‐normally distributed data; the Welch ANOVA with Dunnett T3's multiple comparisons test was used for data that passed the tests for normality but not equal variance. Statistical analyses and graphing were performed using GraphPad Prism (v.9.0, GraphPad Software, La Jolla, CA). Statistical significance was denoted as * (*p* < 0.05), ** (*p* < 0.01), *** (*p* < 0.001), and ****(*p* < 0.0001).

## Conflict of Interest

The authors declare no conflict of interest.

## Author Contributions

C.M., Y.H., and S.Y contributed equally to this work. Y.H., J.Z., G.Z., Y.Z., B.F., and W.Z. conducted the experiments. Y.M., S.Y., L.L., Z.H.L., M.Z., and H.Z. offered advice. X.G., G.F., and C.M. designed the experiments and wrote the paper.

## Supporting information



Supporting Information

## Data Availability

The data that support the findings of this study are available from the corresponding author upon reasonable request.
