# Supplementary material for: Wogonin Attenuates Atherosclerosis via KLF11‐Mediated Suppression of PPARα‐YAP1‐Driven Glycolysis and Enhancement of ABCA1/G1‐Mediated Cholesterol Efflux
Source: Adv Sci (Weinh). 2025 May 21;12(23):2500610. doi: 10.1002/advs.202500610 (PMC12199419; doi:10.1002/advs.202500610)
Supplement: Supplementary file 1 — Supporting Information [file ADVS-12-2500610-s001.docx]

**Supplementary materials**

**Wogonin Attenuates Atherosclerosis via KLF11-Mediated Suppression of PPARα-YAP1-Driven Glycolysis and Enhancement of ABCA1/G1-Mediated Cholesterol Efflux**

**Running title:** Targeting PPARα-KLF11-YAP1 by Wogonin Mitigates Atherogenesis

Chuanrui Ma^1,2,3#^, Yunqing Hua^1,2,3#^, Shu Yang^5*^, Yun Zhao^1^, Wei Zhang^1^, Jing Zhang^1^, Boxuan Feng^1^, Guobin Zheng^6^, Lan Li^1,2^, Zhihao Liu^1,2^, Han Zhang^2^, Mingjun Zhu^7*^, Xiumei Gao^1,2,4*^, Guanwei Fan ^1,2,3,4*^

^1^First Teaching Hospital of Tianjin University of Traditional Chinese Medicine, National Clinical Research Center for Chinese Medicine Acupuncture and Moxibustion, Tianjin, China

^2^State Key Laboratory of Component-Based Chinese Medicine, Tianjin 301617, China

^3^Tianjin Key Laboratory of Translational Research of TCM Prescription and Syndrome

^4^Haihe Laboratory of Modern Chinese Medicine, Tianjin, 300193, China

^5^Department of Endocrinology, Shenzhen People's Hospital, The Second Clinical Medical College of Jinan University & The First Affiliated Hospital of Southern University of Science and Technology, Shenzhen 518020, Guangdong, China

^6^NHC Key Laboratory of Hormones and Development, Tianjin Key Laboratory of Metabolic Diseases, Chu Hsien-I Memorial Hospital & Tianjin Institute of Endocrinology, Tianjin Medical University, Tianjin 300134, China.

^7^Department of Cardiovascular Diseases, The First Affiliated Hospital of Henan University of Traditional Chinese Medicine, Zhengzhou, China.

#These authors contributed equally to this article.

Correspondence should be addressed to:

Guanwei Fan, PhD; Xiumei Gao, PhD; Mingjun Zhu, PhD;

First Teaching Hospital of Tianjin University of Traditional Chinese Medicine, National Clinical Research Center for Chinese Medicine Acupuncture and Moxibustion, Tianjin, China; No.88, Chang Ling Road, Li Qi Zhuang Jie, Xi Qing District, Tianjin, P.R. China;

E-mail: guanwei.fan@tjutcm.edu.cn; gaoxiumei@tjutcm.edu.cn; zhumingjun317@163.com


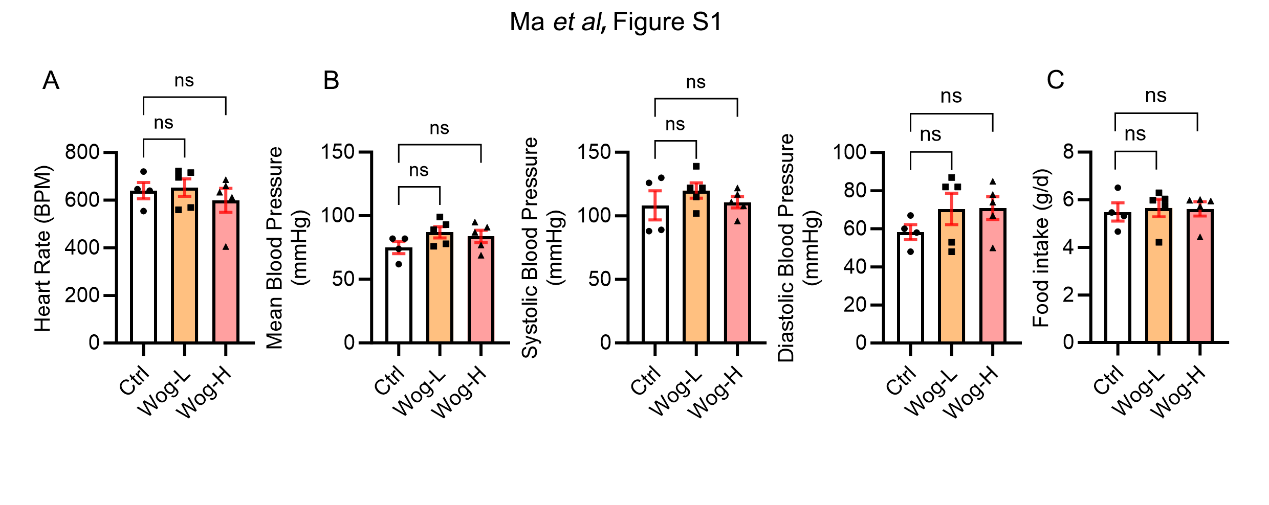


**Figure S1. Figure S1. Wogonin administration does not affect baseline levels of heart rate, blood pressure, or food intake in mice.** C57BL/6 mice were administered varying doses of wogonin for 14 days. Measurements include (**A**) heart rate, (**B**) mean arterial pressure (systolic and diastolic), and (**C**) daily food intake, control n=10, Wog-L and Wog-H n=5. Data are presented as mean ± SEM. P-values are shown in the figure S1 by One-way ANOVA with Tukey's multiple comparisons test. ns: not significantly different.


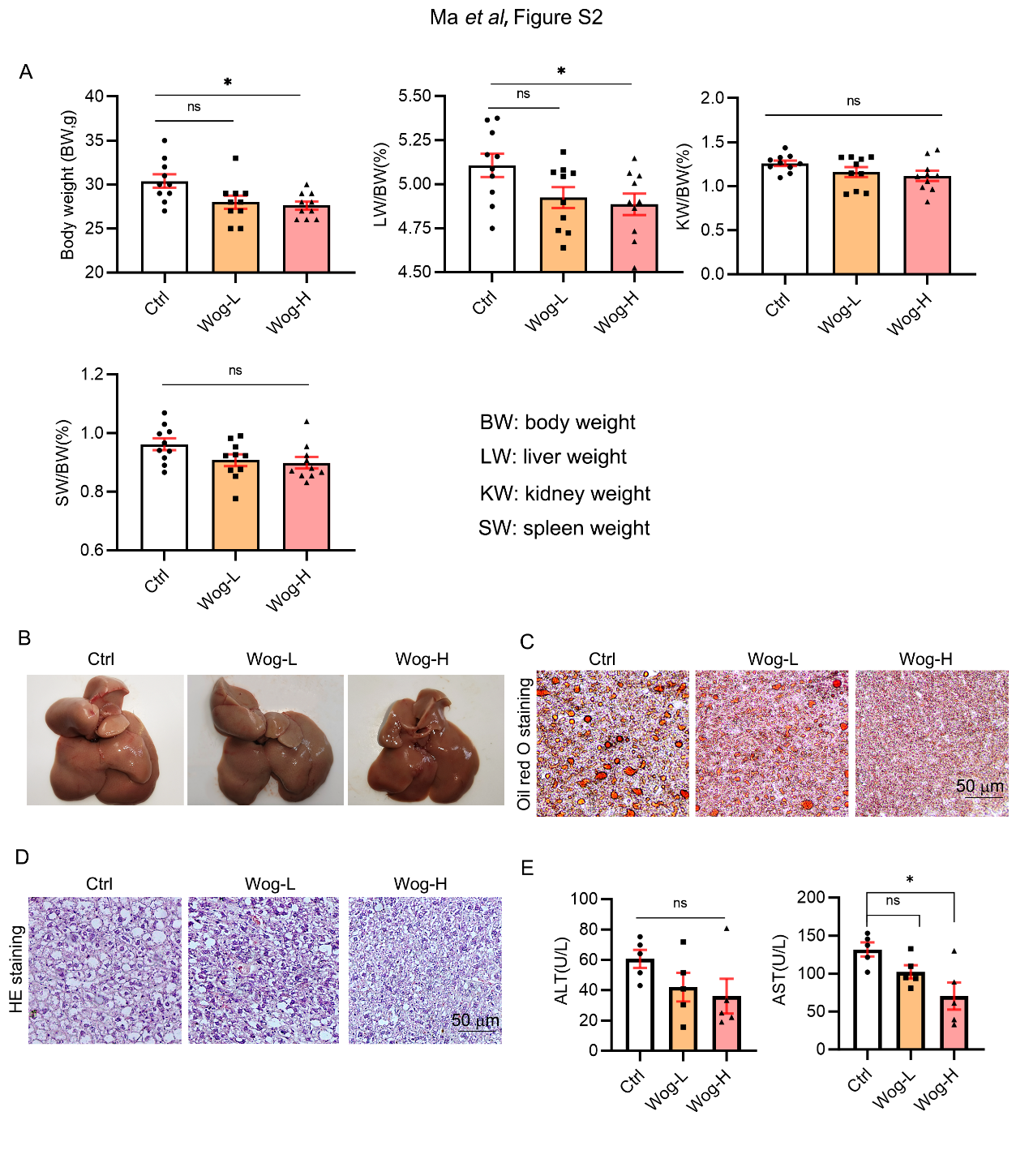


**Figure S2. Effect of wogonin on body weight and organ index in atherosclerotic mice.** Eight-week-old male LDLR^-/-^ mice were fed a Western diet for 16 weeks to form an atherosclerosis model. (**A**) Body weight of LDLR^-/-^ mice in each group as well as organ indices, n=10. Representative images of mouse liver (**B**), Oil red O staining (**C**) and HE staining (**D**) of mouse liver. (**E**) Serum AST and ALT were detected using kits,n=5. Data are presented as mean ± SEM. P-values are shown in the figure S2 by One-way ANOVA with Tukey's multiple comparisons test. *P<0.05 significantly different as indicated; ns: not significantly different.


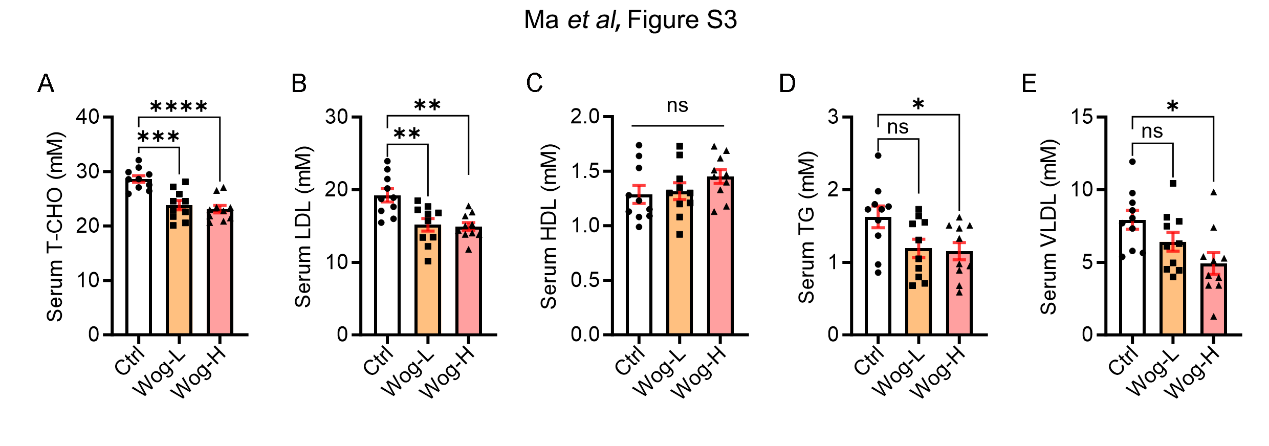


**Figure S3. Wogonin improves the lipid disorder in vivo under HFD conditions.** (**A-E**) Serum levels of total cholesterol (T-CHO), LDL and HDL, VLDL, and TG (mM) were determined by biochemical analysis, n=10. Data are presented as mean ± SEM. P-values are shown in the figure S2 by One-way ANOVA with Tukey's multiple comparisons test. *P<0.01 significantly different as indicated; ns: not significantly different.


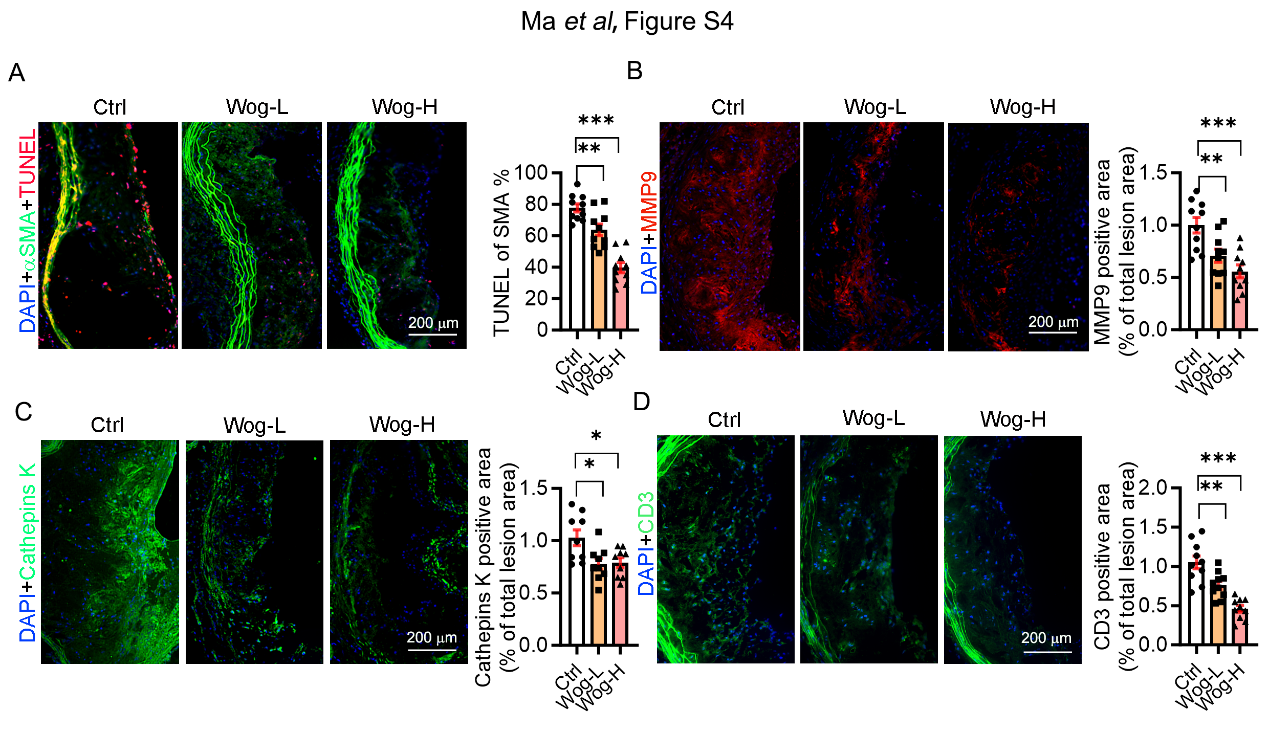


**Figure S4. Wogonin ameliorates the inflammatory microenvironment within atherosclerotic plaques.** Apoptosis of VSMCs (**A**), expression levels of MMP-9 (**B**), cathepsin K (**C**) as well as T-cell infiltration (**D**) within plaques were assessed, n=10. Data are presented as mean ± SEM. P-values are shown in the figure S4 by One-way ANOVA with Tukey's multiple comparisons test. *P<0.05, **P<0.01 and ***P<0.001


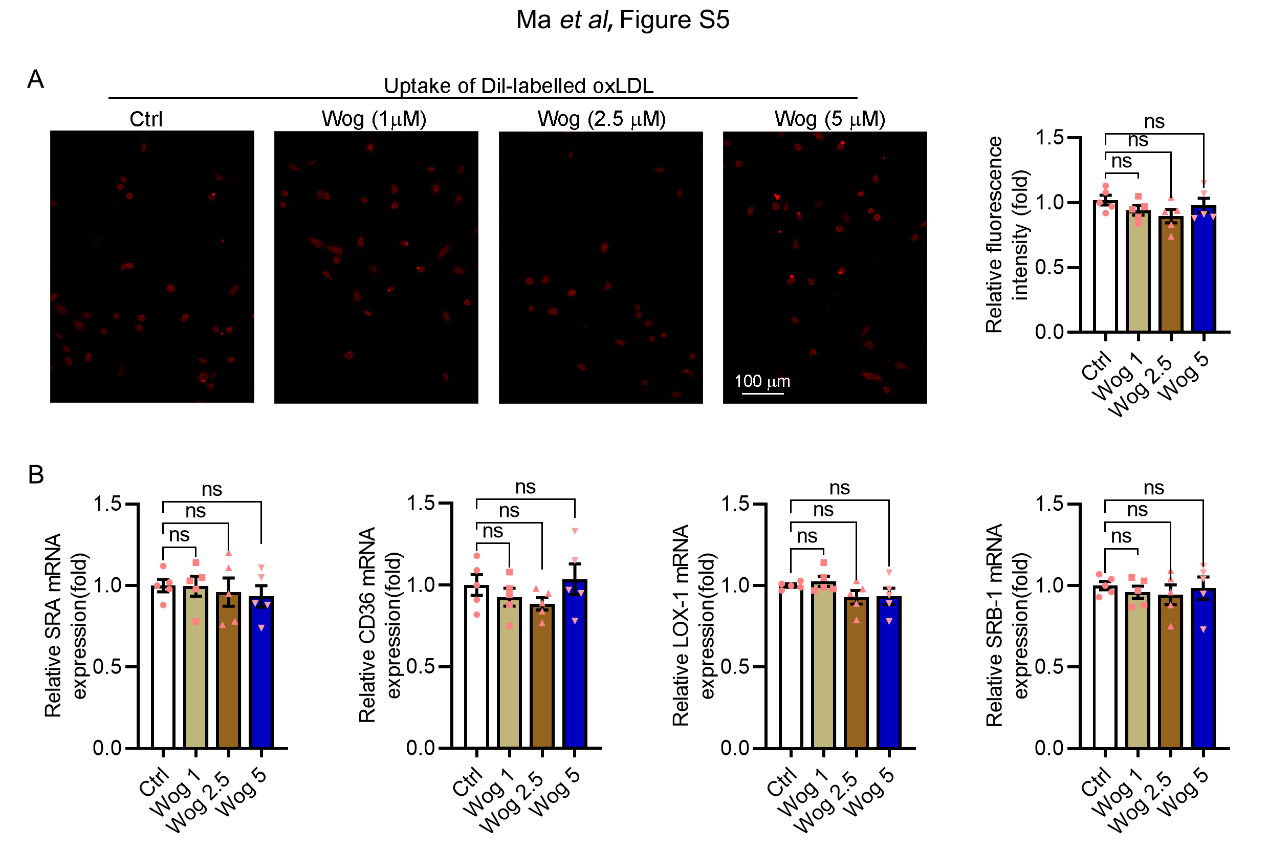


**Figure S5. Wogonin did not affect the uptake of oxLDL and the** **scavenger receptor expression in vitro.** (**A**) Representative images showing uptake of Dil-labelled oxLDL, n=5. (**B**) Relative mRNA expression of scavenger receptors, including SRA, CD36, LOX-1, SRB-1, n=5. Data are presented as mean ± SEM. P-values are shown in the figure S5 by One-way ANOVA with Tukey's multiple comparisons test.ns: not significantly different.


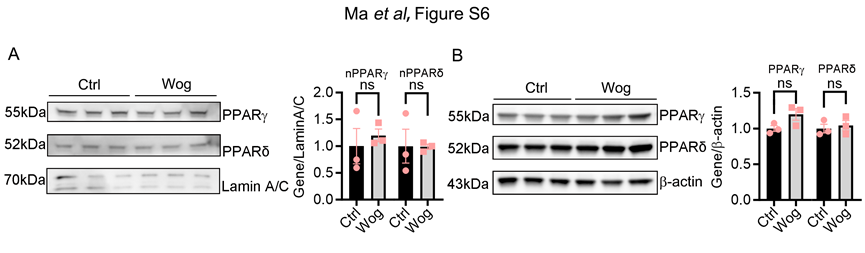


**Figure S6. Wogonin did not affect the expression and nuclear translocation of PPARγ and PPARδin RAW264.7 cells.** (**A, B**) RAW264.7 cells received indicated treatment overnight. Expression of PPARγ and PPARδ in nuclear extract (A) and total cellular extract (B) was determined by Western blot. n=3. Data are presented as mean ± SEM. P-values are shown in the figure S6 by One-way ANOVA with Tukey's multiple comparisons test. ns: not significantly different.


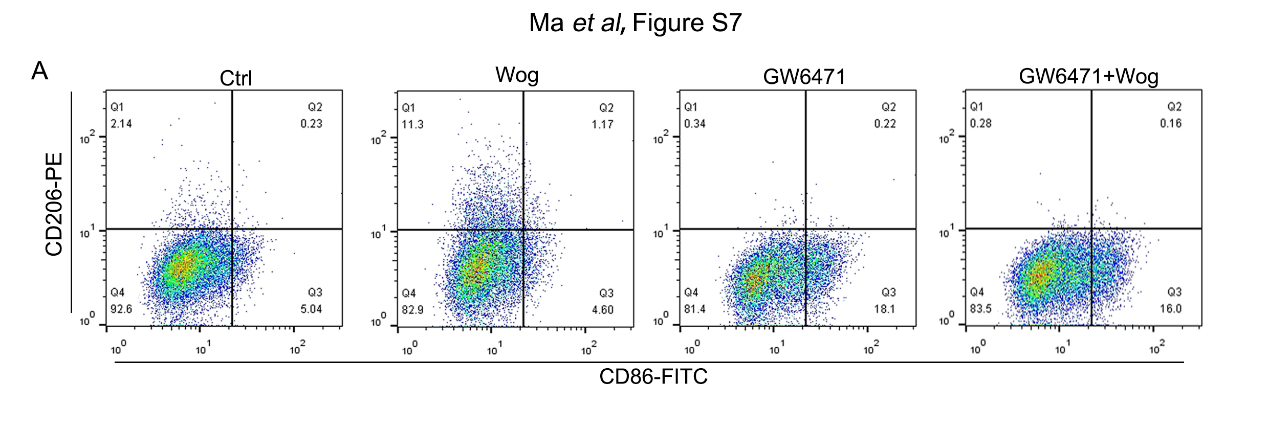


**Figure S7. Wogonin induces macrophage polarization towards the M2 type through PPARα *in vitro*.** (**A**) RAW264.7 cells were incubated with wogonin (5μM) for 16 hours or/and GW6471 (10μM) for 48 hours. The subtypes of RAW264.7 cells were detected by flow cytometry, n=5.


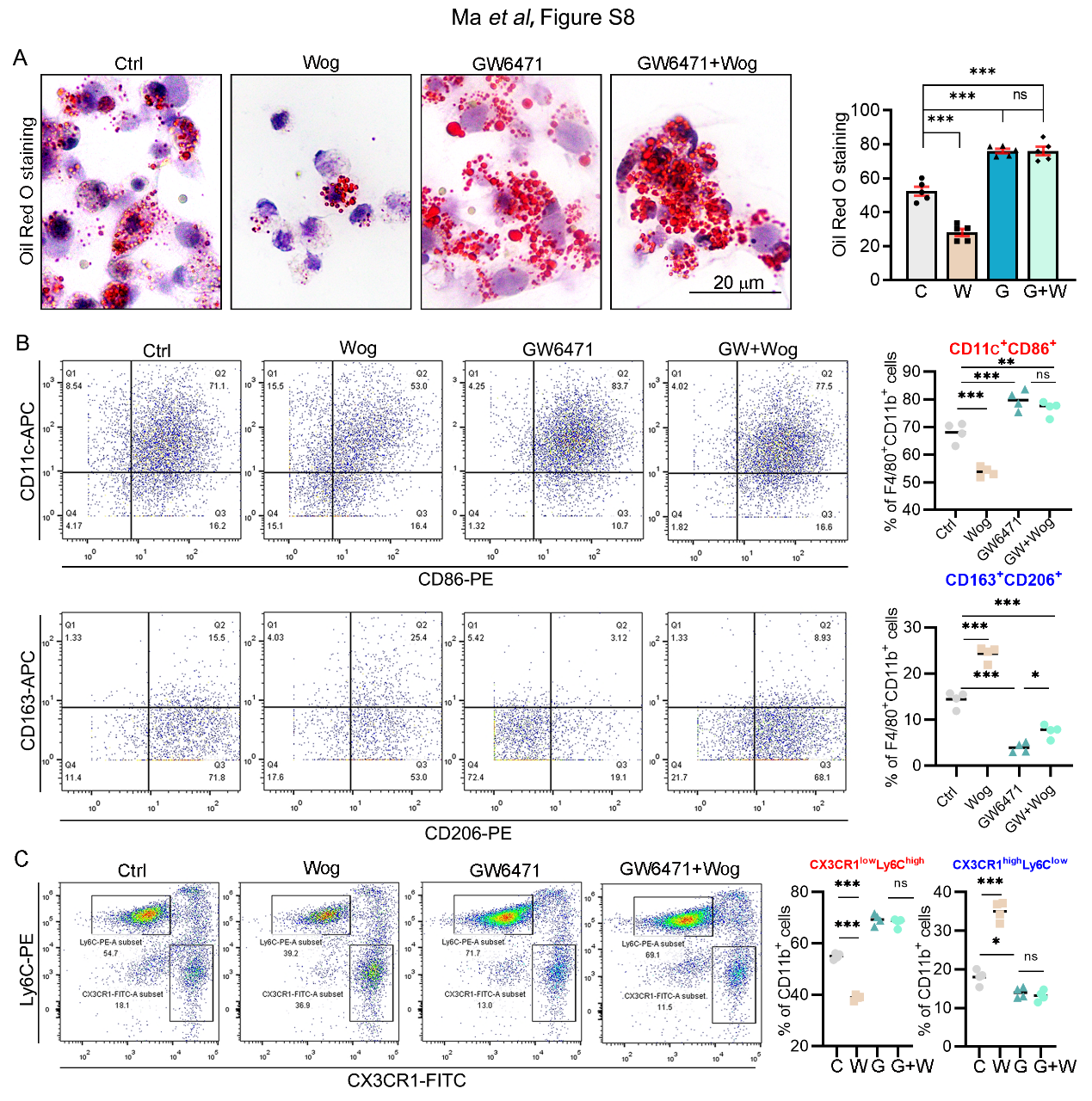


**Figure S8. Inhibition of PPARα promoted macrophage-derived foam cell formation and lead the macrophages and monocytes toward inflammatory status in vivo.** The mice were treated with PPARα inhibitor GW6471 in the presence or absence of wogonin and then the peritoneal and blood were collected for the following assay. (**A**) Oil red O staining on peritoneal macrophages to assess the formation of foam cells, n=5. (**B**) The proportions of M1 and M2 subtypes of peritoneal macrophages were detected by flow cytometry. F4/80 and CD68 were used as primary gates to identify macrophages. Then, CD11b and CD86 were used to mark M1 macrophages; while CD206 and CD163 were used as markers for M2 macrophages, n=4. (**C**) Two subpopulations of the monocytes that were characterized as Ly6C^high^CX3CR1^low^ and Ly6C^low^CX3CR1^high^ were detected by flow cytometry, n=5. Data are presented as mean ± SEM. P-values are shown in the figure (A-C) by One-way ANOVA with Tukey's multiple comparisons test. *P<0.05, **P<0.01 and ***P<0.001, significantly different as indicated; ns: not significantly different.


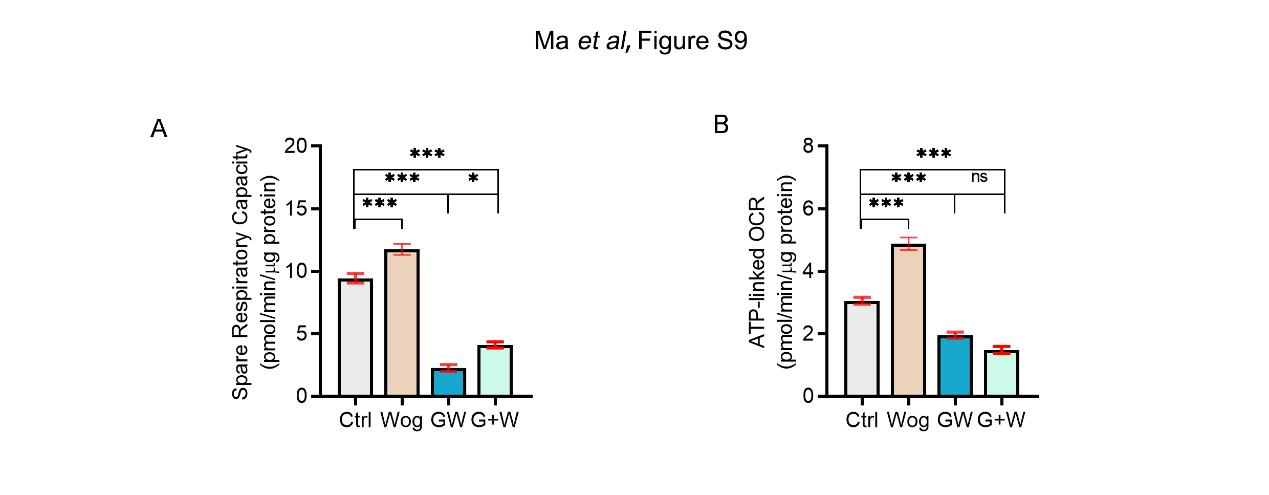


**Figure S9. Wogonin up-regulates cellular spare respiratory capacity and ATP synthesis ability.** The spare respiratory capacity (**A**) and ATP synthesis capacity (**B**) were assessed according to the oxygen consumption rate (OCR) of peritoneal macrophages, normalized by the content of protein in each 96-well sample, n=5. Data are presented as mean ± SEM. P-values are shown in the figure (A and B) by One-way ANOVA with Tukey's multiple comparisons test. *P<0.05 and ***P<0.001, significantly different as indicated; ns: not significantly different.


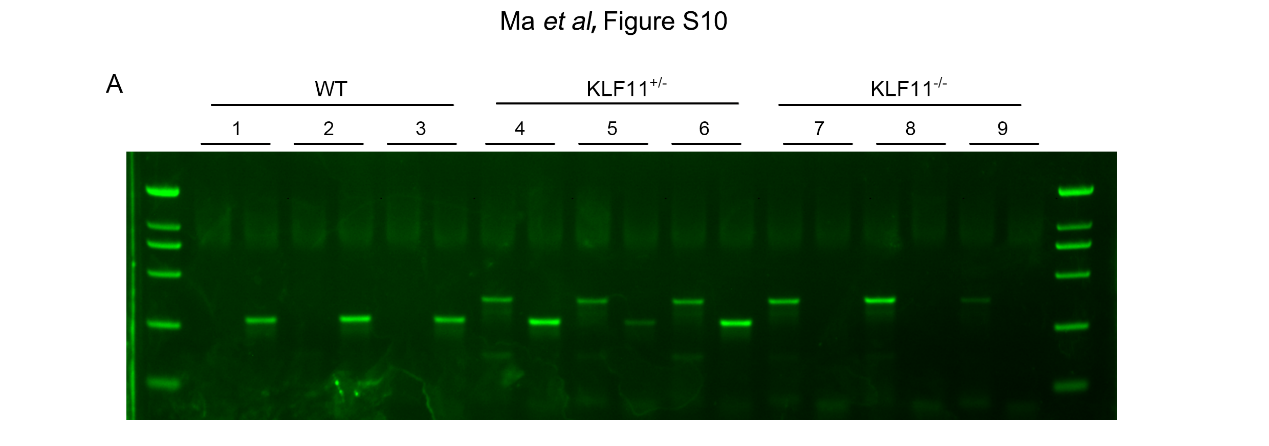


**Figure S10. KLF11 knockout mice were determined by PCR.** (**A**) Identification of KLF11^-/-^ mice by agarose gel electrophoresis. The genotypes of mice numbered 1 to 3, 4 to 6, and 7 to 9 were classified as wild type (WT), KLF11^+/-^, and KLF11^-/-^, respectively.


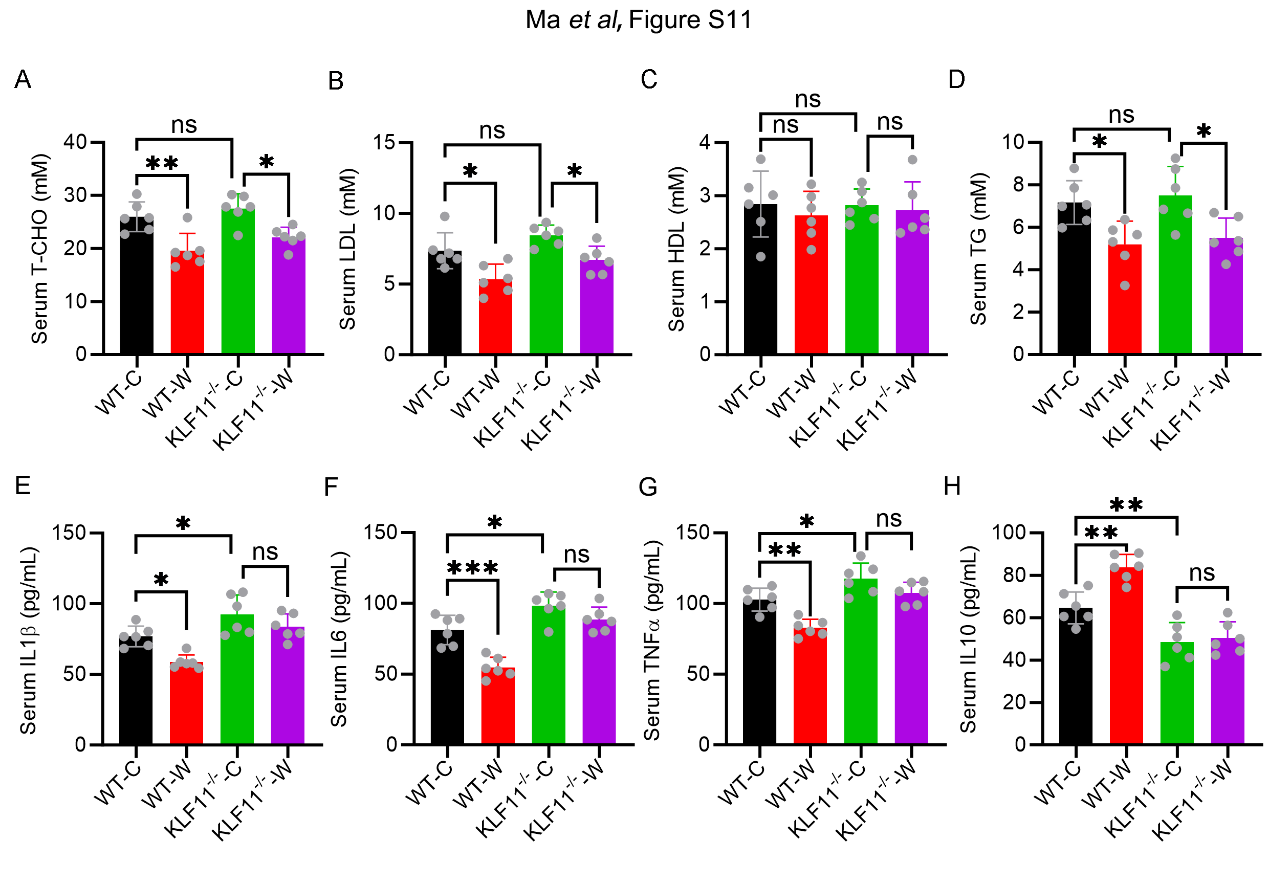


**Figure S11. KLF11 knockout abolished the anti-inflammatory but not the lipid-lowering effect of wogonin.** (**A-H**) 8-week-aged KLF11^−/−^ and WT mice were fed with a 16-week HFD and simultaneously given AAV-PCSK9 (200μL, 3x10^11^ vg/mice) through tail vein injection to construct an atherosclerosis model. After being treated with wogonin for 16 weeks, the mice were euthanized and blood samples were collected for testing lipid and inflammatory cytokines, n=6. Data are presented as mean ± SEM. P-values are shown in the figure (A-H) by One-way ANOVA with Tukey's multiple comparisons test. *P<0.05, **P<0.01 and ***P<0.001, significantly different as indicated; ns: not significantly different.


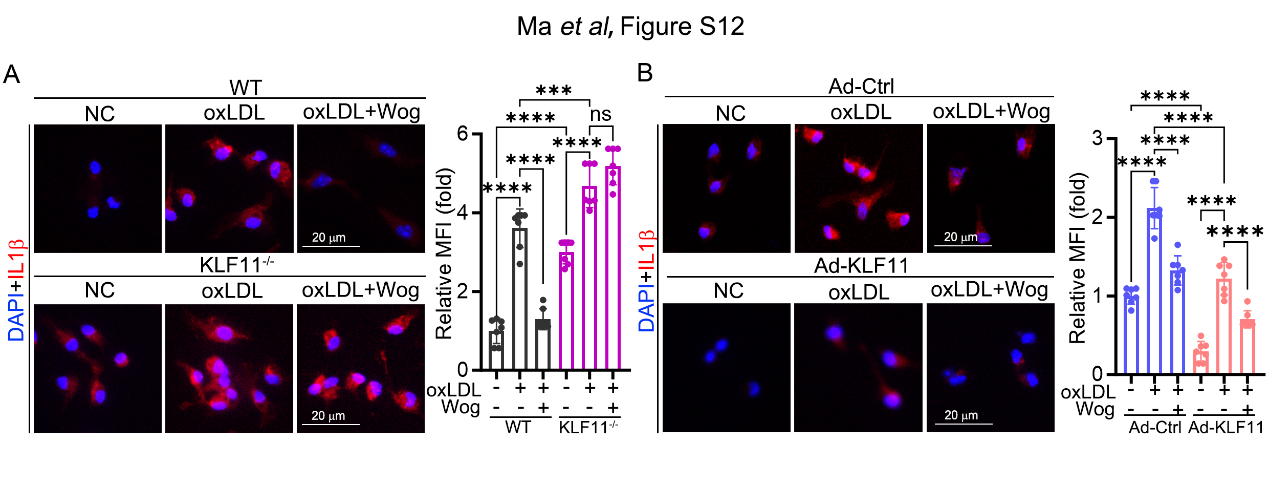


**Figure S12. KLF11 plays an important role in the anti-inflammatory effect of wogonin in vitro.** (**A**) Peritoneal macrophages that were isolated from KLF11^+/+^(WT) and KLF11^-/-^ mice were treated with oxLDL(100 μg/mL) for 24 h in the presence or absence of wogonin (5μM). After incubation, the expression of IL1β was detected by immunofluorescent staining, and the image was taken by an immunofluorescence microscope, n=6. (**B**) Peritoneal macrophages that were isolated from KLF11^+/+^(WT) mice were infected by Ad-KLF11 and then treated with oxLDL(100 μg/mL) for 24 h in the presence or absence of wogonin (5μM). The expression of IL1β was detected by immunofluorescent staining and the image was taken by an immunofluorescence microscope, n=6. Data are presented as mean ± SEM. P-values are shown in the figure (A and B) by One-way ANOVA with Tukey's multiple comparisons test. ***P<0.001, and ****P<0.0001, significantly different as indicated; ns: not significantly different.


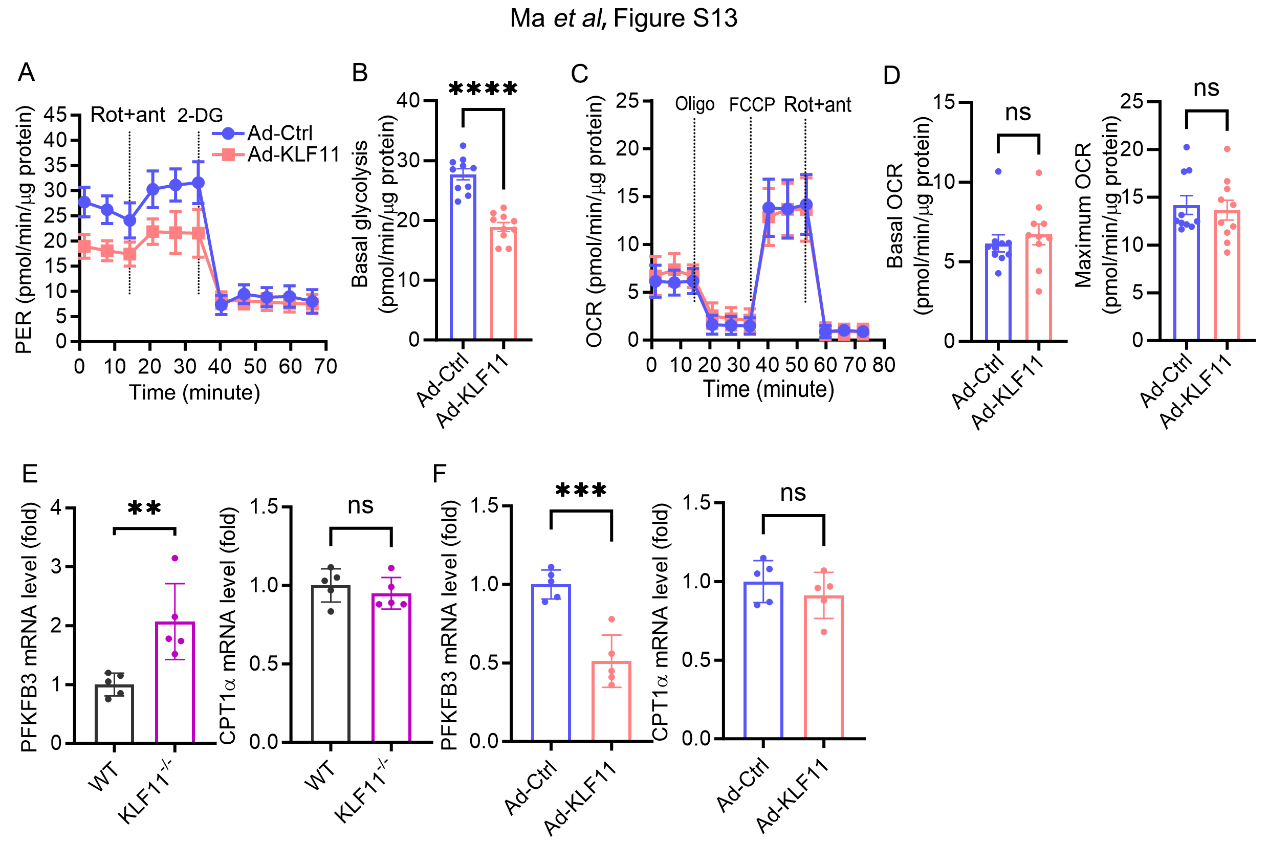


**Figure S13. KLF11 overexpression reduced glycolysis** **without affecting FAO and KLF11 knockout promotes PFKFB3 expression without affecting CPT1α expression in vitro.** (**A, B**) Tracking and quantifying the proton efflux rate (PER) and basal glycolytic capacity of RAW264.7 cells that overexpressed KLF11 by infecting Ad-KLF11, n=10. (**C, D**) PER curves of RAW264.7 cells that overexpressed KLF11 by infecting Ad-KLF11, n=10. (**E, F**) The expression of PFKFB3 and CPT1α in the peritoneal macrophages from KLF11^-/-^ mice or in RAW264.7 cells that overexpressed KLF11 by infecting Ad-KLF11, n=5. Data are presented as mean ± SEM. P-values are shown in the figure (A-F) by One-way ANOVA with Tukey's multiple comparisons test. **P<0.01 and ***P<0.001, and ****P<0.0001, significantly different as indicated; ns: not significantly different.


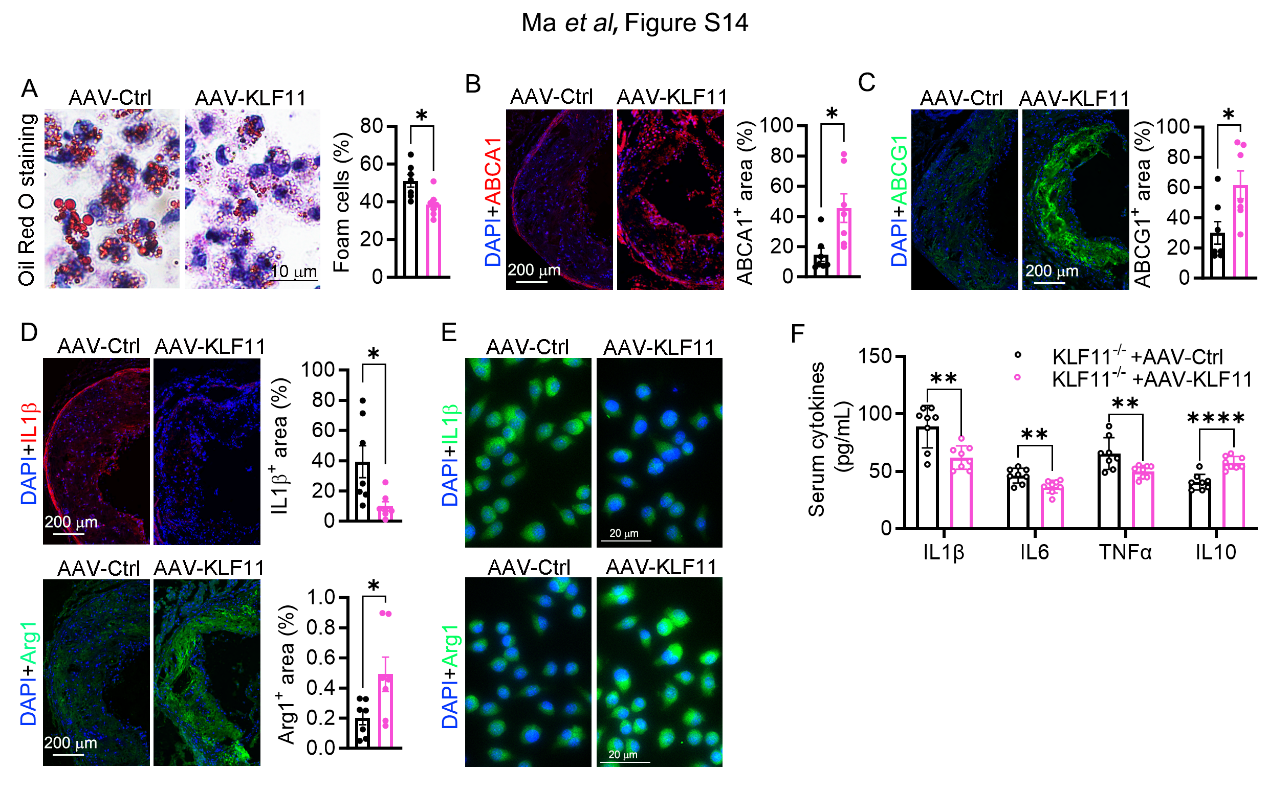


**Figure S14. KLF11 gain-of-function attenuates foam cell formation and cellular inflammation.** KLF11^-/-^ mice were infected with AAV-PCSK9 and fed with HFD to induce the atherosclerosis model and simultaneously infected with AAV-KLF11 to replenish the KLF11. After treatment, the peritoneal macrophages, aorta, and serum were collected for the following assay. (**A**) Oil red O staining on peritoneal macrophages to assess the formation of foam cells, n=7. (**B-D**) Expression of ABCA1, ABCG1, IL1β, and Arg1 in the plaque was determined by immunofluorescent staining, n=7. (**E**) The expression of L1β and Arg1 in the peritoneal macrophages was determined by immunofluorescent staining, n=7. (**F**) The levels of L1β, Arg1, TNFα, and IL10 in the serum were determined by Elisa kit, n=7. Data are presented as mean ± SEM. P-values are shown in the figure (A,B,C,D,F) by One-way ANOVA with Tukey's multiple comparisons test. *P<0.05, **P<0.01, and ****P<0.0001, significantly different as indicated; ns: not significantly different.


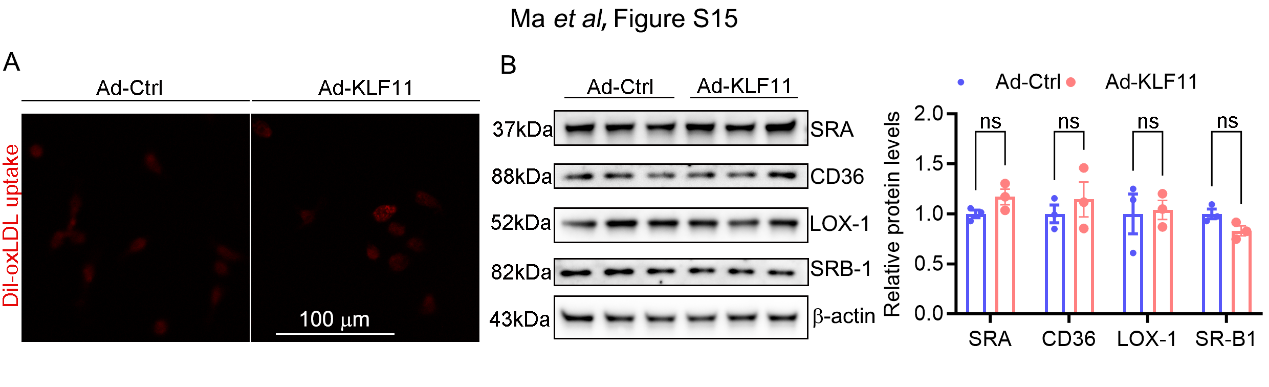


**Figure S15. KLF11 overexpression did not affect the lipid uptake and scavenger expression in vitro.** (**A**) RAW264.7 cells were transfected with Ad-KLF11 and then incubated with Dil-oxLDL. At the end of stimulation, RAW264.7 cells were washed with PBS to remove Dil-oxLDL. The depiction of DiI-oxLDL localization was determined by an immunofluorescence microscope. (**B**) RAW264.7 cells were transfected with Ad-KLF11 and then the expression of scavengers, including SRA, CD36, LOX1, and SRB-1, were detected by western blot, n=3. Data are presented as mean ± SEM. P-values are shown in the figure (B) by One-way ANOVA with Tukey's multiple comparisons test. ns: not significantly different.

**Table S1. The sequences of primers for qRT-PCR analysis**

| Gene | Forward Primer | Reverse Primer |
| --- | --- | --- |
| *Mus IL-1b* | GAAATGCCACCTTTTGACAGTG | TGGATGCTCTCATCAGGACAG |
| *Mus IL-6* | CTGCAAGAGACTTCCATCCAG | AGTGGTATAGACAGGTCTGTTGG |
| *Mus TNFa* | CCTGTAGCCCACGTCGTAG | GGGAGTAGACAAGGTACAACCC |
| *Mus NFkB* | ATGGCAGACGATGATCCCTAC | TGTTGACAGTGGTATTTCTGGTG |
| *Mus INFg* | ATGAACGCTACACACTGCATC | CCATCCTTTTGCCAGTTCCTC |
| *Mus CCL5* | GCTGCTTTGCCTACCTCTCC | TCGAGTGACAAACACGACTGC |
| *Mus CCL2* | TAAAAACCTGGATCGGAACCAAA | GCATTAGCTTCAGATTTACGGGT |
| *MusNLRP3* | ATCAACAGGCGAGACCTCTG | GTCCTCCTGGCATACCATAGA |
| *Mus IL-12* | ATGGAGTCATAGGCTCTGGAAA | CCGGAGTAATTTGGTGCTTCAC |
| *Mus VCAM* | AGTTGGGGATTCGGTTGTTCT | CCCCTCATTCCTTACCACCC |
| *Mus iNOS* | GTTCTCAGCCCAACAATACAAGA | GTGGACGGGTCGATGTCAC |
| *Mus MMP7* | TCGCAAGGAGAGATCATGGAG | CTGCGTCCTCACCATCAGTC |
| *Mus TGFb* | CTTCAATACGTCAGACATTCGGG | GTAACGCCAGGAATTGTTGCTA |
| *Mus IL-4* | ATCATCGGCATTTTGAACGAGG | TGCAGCTCCATGAGAACACTA |
| *Mus IL-4* | TGTCCCTAATGACAGCTCCTT | GCATCCACCCAAATGACACAT |
| *Mus CPT1a* | GCCCATGTTGTACAGCTTCC | TTGGAAGTCTCCCTCCTTCA |
| *Mus PFKFB3* | GATCTGGGTGCCCGTCGATCACCG | CAGTTGAGGTAGCGAGTCAGCTTC |
| *Mus Arg* | TGTCCCTAATGACAGCTCCTT | GCATCCACCCAAATGACACAT |
| *Mus b-actin* | ATGGAGGGGAATACAGCCC | TTCTTTGCAGCTCCTTCGTT |
| *Hom IL-1b* | ATGATGGCTTATTACAGTGGCAA | GTCGGAGATTCGTAGCTGGA |
| *Homo TNFa* | CCTCTCTCTAATCAGCCCTCTG | GAGGACCTGGGAGTAGATGAG |
| *Homo CCL5* | CCAGCAGTCGTCTTTGTCAC | CTCTGGGTTGGCACACACTT |
| *Homo VCAM1* | TTTGACAGGCTGGAGATAGACT | TCAATGTGTAATTTAGCTCGGCA |
| *Homo ICAM1* | ATGCCCAGACATCTGTGTCC | GGGGTCTCTATGCCCAACAA |
| *Homo VEGF* | AGGGCAGAATCATCACGAAGT | AGGGTCTCGATTGGATGGCA |
| *Homo eNOS* | TGATGGCGAAGCGAGTGAAG | ACTCATCCATACACAGGACCC |
| *Homo b-actin* | CTGGAACGGTGAAGGTGACA | AAGGGACTTCCTGTAACAATGCA |

**Table S2. Complete blood count results in C57BL/6 mice^†^**

| Treatment | Ctrl | Wog-L | Wog-H |
| --- | --- | --- | --- |
| WBC (×10^9^/L) | 2.73 ± 1.31 | 3.24 ± 0.97 | 2.56 ± 0.43 |
| Neutrophils (×10^9^/L) | 0.42 ± 0.3 | 0.74 ± 0.52 | 0.42± 0.17 |
| Lymphocytes (×10^9^/L) | 2.21 ± 0.99 | 2.31 ± 0.48 | 2.07± 0.45 |
| Monocytes (×10^9^/L) | 0.08 ± 0.07 | 0.16 ± 0.14 | 0.06 ± 0.01 |
| RBC (×10^12^/L) | 7.82 ± 2.78 | 9.18 ± 0.6 | 9.39 ± 0.38 |
| Hemoglobin (g/L) | 126.25 ± 44.43 | 148.8 ± 8.42 | 152 ± 6.51 |
| Hematocrit (%) | 36.85 ± 13.18 | 43.66 ± 2.53 | 43.84 ± 1.6 |
| MCV (fL) | 47.03 ± 0.33 | 47.56 ± 0.38 | 46.7 ± 0.4 |
| MCH (pg) | 16.15 ± 0.15 | 16.24 ± 0.22 | 16.18 ± 0.24 |
| MCHC (g/L) | 343.25 ± 4.44 | 341.2 ± 2.48 | 346.4 ± 4.27 |
| RDW-CV (%) | 15.63 ± 1.43 | 14.34 ± 1.02 | 14.94 ± 1.7 |
| RDW-SD (fL) | 32.55 ± 3.38 | 29.78 ± 1.95 | 30.82 ± 3.65 |

†:C57BL/6 mice were administered varying doses of wogonin for 14 days, followed by whole blood collection for complete blood count analysis. Dates are presented as mean ± SD (Control n=4, Wog-L and Wog-H n=5)
